# Advances in Fecal Microbiota Transplantation for Gut Dysbiosis‐Related Diseases

**DOI:** 10.1002/advs.202413197

**Published:** 2025-02-27

**Authors:** Shuna Hou, Jiachen Yu, Yongshuang Li, Duoyi Zhao, Zhiyu Zhang

**Affiliations:** ^1^ Department of Orthopedics The Fourth Affiliated Hospital of China Medical University China Medical University Liao Ning Shen Yang 110032 P. R. China; ^2^ Department of general surgery The Fourth Affiliated Hospital of China Medical University China Medical University Liao Ning Shen Yang 110032 P. R. China

**Keywords:** clinical efficacy, Clostridioides difficile infection, fecal bacteria transplantation, intestinal flora, intestinal diseases

## Abstract

This article provides an overview of the advancements in the application of fecal microbiota transplantation (FMT) in treating diseases related to intestinal dysbiosis. FMT involves the transfer of healthy donor fecal microbiota into the patient's body, aiming to restore the balance of intestinal microbiota and thereby treat a variety of intestinal diseases such as recurrent Clostridioides difficile infection (rCDI), inflammatory bowel disease (IBD), constipation, short bowel syndrome (SBS), and irritable bowel syndrome (IBS). While FMT has shown high efficacy in the treatment of rCDI, further research is needed for its application in other chronic conditions. This article elaborates on the application of FMT in intestinal diseases and the mechanisms of intestinal dysbiosis, as well as discusses key factors influencing the effectiveness of FMT, including donor selection, recipient characteristics, treatment protocols, and methods for assessing microbiota. Additionally, it emphasizes the key to successful FMT. Future research should focus on optimizing the FMT process to ensure long‐term safety and explore the potential application of FMT in a broader range of medical conditions.

## Graphical abstract

This article reviews the use of fecal microbiota transplantation (FMT) to treat intestinal diseases like recurrent Clostridioides difficile infection (rCDI), inflammatory bowel disease (IBD), and irritable bowel syndrome (IBS). It discusses FMT's efficacy, influencing factors such as donor selection, recipient characteristics, and treatment protocols while emphasizing the need for further research to optimize safety and explore broader applications.

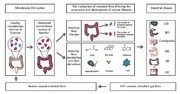

## 1 Introduction

Within the gastrointestinal tract, there exist over a billion varieties of microbiota to ensure the normal functioning of the digestive system, encompassing known and unknown bacteria, fungi, protists, and viruses, collectively shaping a dynamic equilibrium within the gut, known as the microbiome.^[^ ^1^ ^]^ This equilibrium demonstrates relative stability and a certain resilience.^[^ ^2^ ^]^ Primarily dominated by bacteria, the indigenous flora in the adult gut comprises four major phyla: Firmicutes, Bacteroidetes, Actinobacteria, and Proteobacteria.^[^ ^3^, ^4^, ^5^ ^]^ Over the past few decades, there has been a growing recognition of the significance of the gut microbiota for human health and well‐being. A healthy and diverse gut microbiota plays a crucial role in assisting digestion, promoting metabolism, regulating the immune system, and inhibiting bacterial growth, among other functions,^[^ ^6^, ^7^ ^]^ essential for the normal functioning of the human body. However, driven by host and environmental factors, alterations in the composition and functionality of the gut microbiota can occur. The composition of the gut microbiota undergoes significant changes, characterized by a reduction in beneficial bacteria and an overgrowth of pathogenic species. This dysbiosis (ecological imbalance) disrupts the intestinal barrier, activates the immune system, and triggers chronic inflammation.^[^ ^8^, ^9^, ^10^ ^]^ Specifically, the reduction of *Lactobacillus* and *Bifidobacterium*, the depletion of *Clostridia* groups, and the overgrowth of pathogenic bacteria such as *Clostridioides difficile* and *Escherichia coli* are closely associated with various gastrointestinal disorders, including constipation, irritable bowel syndrome, and recurrent *Clostridioides difficile* infections.^[^ ^11^, ^12^ ^]^ Restoring microbial diversity and reintroducing healthy microbiota (e.g., through FMT) can effectively modulate the gut microbiome and alleviate diseases caused by dysbiosis.Though the causal relationship between microbial variations and the pathophysiological processes of most diseases remains incompletely understood and whether they solely result from disease progression remains inconclusive, it is beyond doubt that by modulating the gut microbiota and restoring its balance and diversity, there may be potential benefits in treating or preventing diseases associated with microbial dysbiosis.

**Figure 1:**
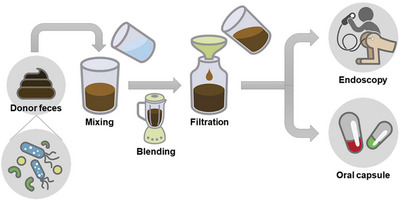
A simplified flowchart of FMT. The fecal sample from a healthy donor undergoes processes such as blending, stirring, and filtration, and is subsequently transplanted into the gastrointestinal tract of a recipient either via endoscopy or capsule.

Fecal Microbiota Transplantation(FMT), also called “feces transplantation,” “human intestinal microbiota transfer” and “fecal bacteriotherapy”, is a treatment method that involves mixing, blending, and filtering fecal samples from healthy volunteers, and then transplanting them into the digestive tract of another individual through endoscopy or capsules^[^ ^13^ ^]^ (**Figure** **1**); the objective is to reconstruct damaged or imbalanced gut microbiota, promote diversity and balance of intestinal microbes, and thereby improve certain gut‐related diseases^[^ ^14^ ^]^; although FMT is a relatively new medical term, its concept and practice can be traced back to ancient times. The earliest documented use of fecal suspension therapy dates back to at least 1700 years ago in traditional Chinese medicine^[^ ^15^ ^]^; the renowned traditional Chinese medicine practitioner Ge Hong mentioned in his writings the use of a so‐called “yellow soup” to treat food poisoning and severe diarrhea, which was actually a mixture of feces. By the 16th century, another Chinese doctor, Li Shizhen, documented various fecal preparations in his writings, used to treat a range of gastrointestinal disorders including constipation, fever, vomiting, and pain. In modern history, during World War II, the Bedouins in Africa advised German soldiers stationed in Africa to consume fresh camel feces as a treatment for bacterial dysentery.^[^ ^16^ ^]^ Although primitive, this practice demonstrated the concept of using microbial treatment from feces for diseases. It was not until the early 20th century that advances in microbiology provided a scientific basis for understanding fecal therapy. In 1907, Metchnikoff proposed the potential benefits of microbes on health, but it was not until 1958 that Dr. Ben Eiseman, an American surgeon, first described a case of using fecal enemas to treat pseudomembranous enterocolitis in medical literature, marking the first documented record of FMT in modern medicine.^[^ ^17^ ^]^

FMT, as an emerging therapeutic approach, has demonstrated significant clinical efficacy in recent years, particularly in the treatment of recurrent Clostridioides difficile infection (rCDI). Numerous studies have shown that FMT yields a high clinical cure rate in treating rCDI, with some reports indicating a cure rate of over 80%.^[^ ^18^ ^]^ As FMT continues to be widely adopted in this field, it has gradually become a recommended treatment option. Furthermore, the potential therapeutic effects of FMT in conditions related to dysbiosis, such as irritable bowel syndrome (IBS) and inflammatory bowel disease (IBD), have become a focal point of current research.^[^ ^19^, ^20^, ^21^ ^]^

However, despite the notable efficacy of FMT in certain gastrointestinal disorders, its clinical application continues to face a series of challenges. One of the primary obstacles is the lack of standardization in treatment protocols, which hinders the widespread use of FMT. The variation in disease types, individual patient differences, and diverse treatment regimens result in significant variability in FMT outcomes. For instance, the effectiveness of FMT in treating gastrointestinal diseases is not only influenced by the type of condition but is also closely tied to individual patient factors.^[^ ^19^, ^20^ ^]^ Additionally, the therapeutic process is affected by multiple variables, including pre‐treatment preparation,^[^ ^22^, ^23^ ^]^ the number and volume of fecal infusions,^[^ ^24^, ^25^ ^]^ and donor screening criteria.^[^ ^23^ ^]^ Research has shown that factors such as donor selection, the fecal infusion protocol, and the number of treatments significantly impact the success of the therapy.

Moreover, the long‐term safety of FMT remains inadequately defined. While the short‐term therapeutic outcomes of FMT are promising, further research is required to assess its long‐term safety. Some studies have reported potential risks of infection transmission, particularly when donor screening and testing protocols are insufficient, which may lead to severe adverse effects.^[^ ^26^, ^27^ ^]^ Therefore, establishing stricter donor selection criteria and long‐term safety monitoring mechanisms has become one of the critical issues to address for the clinical application of FMT.

To overcome these challenges, researchers and clinicians have proposed several strategies. First, the use of high‐throughput sequencing technologies to conduct detailed analyses of the gut microbiome can help effectively identify healthy and stable donors. The health status of donors should not only ensure the absence of infectious risks but also maintain the diversity and stability of their gut microbiota to maximize the therapeutic efficacy of FMT. Additionally, standardization of treatment protocols is key to the successful implementation of FMT. Establishing unified treatment guidelines—such as the number of infusions, volume of fecal material, pre‐treatment preparations, and dietary adjustments—can further enhance treatment outcomes.^[^ ^22^, ^23^, ^24^, ^25^ ^]^

The diversification of administration routes for FMT has also been considered an effective strategy to improve efficacy. For example, in addition to the traditional enema approach, oral FMT capsules have been shown to be equally effective in certain patients, offering greater convenience and higher patient compliance. This new administration route opens up additional possibilities for the broader application of FMT.^[^ ^28^ ^]^

Future research should focus on further standardizing FMT treatment protocols, particularly by refining key factors such as donor selection, treatment regimens, and infusion frequency. Furthermore, with the continued advancement of gut microbiology, the potential therapeutic applications of FMT are likely to expand. FMT may not only be limited to treating gastrointestinal disorders but could also hold promise in the management of metabolic diseases, immune system disorders, and even neurological conditions such as autism and Parkinson's disease. By optimizing treatment methods, assessing long‐term safety, and exploring new therapeutic areas, FMT has the potential to become a crucial therapeutic tool for a wide range of diseases in the future.

The purpose of this review is to elucidate the potential pathogenic mechanisms underlying gut microbiota dysbiosis, offering a better understanding of how FMT, by restoring damaged or imbalanced gut microbiota, promotes microbiome diversity and balance, thus improving certain gut‐related diseases. Additionally, this review summarizes the current evidence supporting FMT as a therapeutic approach and explores the key factors influencing its success.

## 2 The Gut Microbiota Serves as a Therapeutic Target

**Figure 2:**
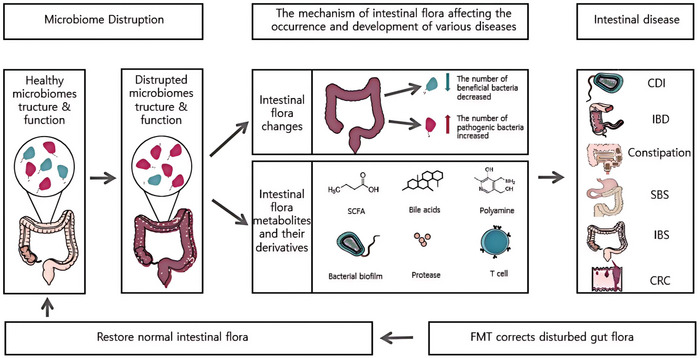
The Mechanism of Gut Microbiota Dysbiosis in Disease Development and the Role of Fecal Microbiota Transplantation (FMT). This diagram illustrates how gut microbiota dysbiosis, by altering the structure, function, and metabolites of the gut microbiota, influences the onset and progression of various diseases, as well as the role of FMT in restoring gut microbiota homeostasis. a). Gut Microbiota Structure and Function in Health: Under normal conditions, the gut microbiota maintains a balanced and diverse ecosystem, with beneficial microorganisms predominating. This balance supports intestinal barrier function and immune modulation. b). Microbial Dysbiosis: When the equilibrium of the microbiota is disrupted (due to factors such as infections, antibiotic use, or poor diet), there is a reduction in beneficial microbes and an increase in pathogenic bacteria. This imbalance leads to a decline in microbiota diversity and impaired gut barrier function. c). i): Mechanistic Impacts: Changes in Gut Microbiota Composition: A decrease in beneficial microbes (indicated by green symbols) and an increase in pathogenic bacteria (indicated by pink symbols) result in dysbiosis. ii): Altered Gut Metabolites and Derivatives: Changes in metabolites and immune factors, such as short‐chain fatty acids (SCFAs), bile acids, polyamines, bacterial biofilms, proteases, and T cells, collectively affect gut homeostasis and systemic health. d)Associated Diseases: Gut microbiota dysbiosis has been linked to the development of various diseases, including Clostridioides difficile infection (CDI), inflammatory bowel disease (IBD), constipation, short bowel syndrome (SBS), irritable bowel syndrome (IBS), and colorectal cancer (CRC). e) i): Role of FMT: Microbiota Restoration: By transplanting the fecal microbiota from a healthy donor, FMT can correct microbial imbalances, restoring the structure and function of the gut microbiota. ii: Disease Treatment: FMT has been shown to improve diseases associated with dysbiosis, particularly demonstrating significant efficacy in the treatment of Clostridioides difficile infection (CDI). f)Future Directions: The diagram illustrates the potential of FMT through arrows, suggesting that restoring the gut microbiota, may improve disease states and promote overall gut health.

The current dysbiosis of the gut microbiota is not merely a manifestation of disease states, but rather a potentially crucial driving factor in the progression of diseases, a notion that remains incompletely elucidated. However, studies suggest that alterations in the structures of specific bacterial species may be one of the pathogenic mechanisms, and dysbiosis can disrupt various metabolic pathways,^[^ ^29^, ^30^, ^31^, ^32^ ^]^ leading to the production of diverse metabolites and their derivatives. These metabolites play a critical role in immune responses and mechanical functions both inside and outside the intestines. Through FMT, these imbalances may be rectified, as illustrated in **Figure** **2**.

### 2.1 Changes in the Structure of the Intestinal Microbiota

A growing body of evidence indicates that changes in the gut microbiota are significantly involved in the development of gastrointestinal disorders. Inflammation, which is a key process in the pathogenesis of various diseases, acts as a natural defense mechanism of the immune system in response to infection, tissue damage, or other abnormal conditions. When inflammation continues or becomes excessively activated, it can serve as a driving force in the progression of disease.^[^ ^33^ ^]^ Given the important role of the gut microbiota in the immune system, an imbalance in the microbial community can hinder proper immune development by depriving beneficial bacteria, while promoting the proliferation of pathogenic bacteria and inflammatory metabolites. Thus, it can be inferred that changes in the gut microbiota contribute significantly to the occurrence of various inflammatory disorders.^[^ ^8^, ^9^, ^34^ ^]^

The gut microbiota can be classified into three categories based on their functions: those that inhibit the development of diseases, those that promote the development of diseases, and those that are harmless to the host under normal circumstances, broadly referred to as probiotics, pathogens, and opportunistic pathogens, respectively. However, some bacteria can switch roles depending on the conditions. Given the complex roles of different microbial populations in various diseases, the following discussion will briefly describe the role of specific microbial populations in specific diseases, and how these populations are influenced by FMT (**Table** **1**).

**Table 1:** Impact of Gut Microbiota on Diseases and Their Mechanisms.

Microbial population	Role and function in disease	Ref.
Probiotics	Immune Modulation: Enhances immune defense, improves gut barrier function, suppresses pathogen growth.	[35]
	Anti‐tumor Properties: Produces siderophores that inhibit tumor progression through the JNK signaling pathway.	[36, 37]
	Clinical Benefits: Alleviates depression and gastrointestinal symptoms in IBS, enhances skin health.	[38, 39]
Pathogens	Immune Evasion: Forms biofilms that protect from antibiotics and immune responses, enhances virulence.	[29, 41]
	Pro‐inflammatory Effects: Triggers inflammatory cytokines leading to chronic inflammation and gut barrier damage.	[29, 42]
	Gut Permeability and Translocation: Damages intestinal mucosa increasing permeability and systemic infections.	[42, 43]
Biofilms	Antibiotic Resistance: Provides a barrier against antibiotics, contributes to resistant infections.	[41]
	Immune Evasion: Hinders immune cell activity, enhancing pathogen survival and disease progression.	[45]
	Disease Progression: Linked with progression of diseases like CRC and IBD, indicates pathogenic infections.	[46]
FMT Effects	Restoring Probiotics: Promotes beneficial bacteria, enhancing gut health and immune functions.	[40]
	Suppressing Pathogens: Reduces pathogenic bacteria, decreases inflammation, restores gut function.	[44]
	Disrupting Biofilms: Breaks down biofilms, improving immune response and treatment effectiveness.	[44]

#### 2.1.1 Role and Function of Probiotics

Probiotics are beneficial microorganisms in the gut that contribute to human health, particularly in alleviating gastrointestinal disorders. Probiotics exert positive effects through various mechanisms, such as enhancing immune defense, improving gut barrier function, and suppressing the growth of pathogenic microorganisms.

##### Immune Modulation

**Figure 3:**
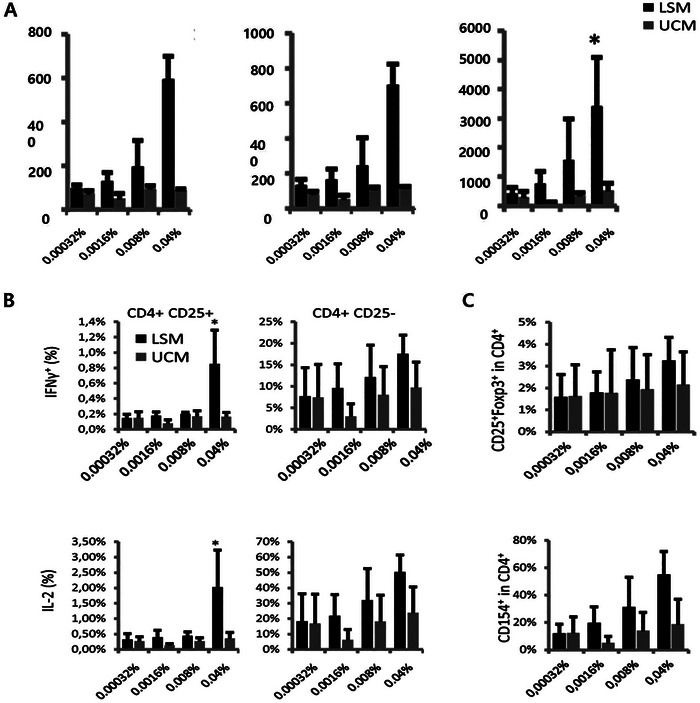
Effects of LSM and UCM on Immune Cell Function under Different Concentrations. This figure demonstrates the impact of Lactic acid bacteria Soluble Medium (LSM) and Unconditioned Medium (UCM) on immune cell activity and function under varying concentrations, divided into three parts (A–C): A) Cell Proliferation Analysis: At different concentrations (ranging from 0.00032% to 0.04%), the proliferation levels of cells in the LSM group (black bars) and the UCM group (gray bars) were assessed. The LSM group exhibited significantly higher proliferative activity at the high concentration (0.04%), indicated by an asterisk (“*”), whereas the cell proliferation in the UCM group remained relatively low. B) CD4^+^ T Cell Subgroup Function: CD4^+^ CD25^+^ T Cells (Left Panel): The expression percentages of IFN‐γ and IL‐2 were significantly higher in the LSM‐treated group, particularly at the high concentration (0.04%), marked with an asterisk (“*”), suggesting that LSM stimulated the activation of effector T cells. CD4^+^ CD25^−^ T Cells (Right Panel): No significant differences were observed between the LSM and UCM groups in terms of IFN‐γ and IL‐2 expression, indicating that the resting CD4^+^ T cells were not significantly affected. C) Regulatory T Cells (Treg) and CD154^+^ T Cells: CD25^+^Foxp3^+^ Tregs (Upper Panel): The proportion of Treg cells did not show significant differences between the LSM and UCM groups across different concentrations. CD154^+^ T Cells (Lower Panel): The proportion of CD154^+^ T cells slightly increased in the LSM group, but the difference did not reach statistical significance. Reproduced with permission.^[^ ^35^ ^]^ Copyright 2018, Frontiers Media S.A.

Probiotics, particularly lactic acid bacteria, modulate immune responses by enhancing the function of dendritic cells (DCs), which play a crucial role in initiating T cell responses. The effects of lactic acid bacteria extend beyond the activation of immune cells; they also involve the regulation of intestinal immune tolerance and the maintenance of intestinal barrier integrity. Ludwig et al.^[^ ^35^ ^]^ proposed that Lactic acid bacteria Soluble Medium (LSM) significantly enhances the ability of dendritic cells to induce cytokine secretion (IFN‐γ and IL‐2) from CD4+CD25+ T cells (**Figure** **3**B), while also increasing the expression of Foxp3+ regulatory T cells (Figure 3A,C). This suggests that LSM, by enhancing T cell reactivity, promotes the role of dendritic cells in immune responses, thereby maintaining immune balance in the gut and preventing excessive immune reactions, which in turn helps to thwart the progression of inflammatory bowel disease and autoimmune disorders. Additionally, through this immunoregulatory mechanism, lactic acid bacteria not only strengthen immune defense in the gut but also improve the integrity of the intestinal barrier, inhibiting pathogen colonization and thereby maintaining the healthy balance of the gut microbiota.

##### Anti‐Tumor Properties

Probiotics also have anti‐cancer effects. Konishi et al.^[^ ^36^ ^]^ found that Lactobacillus casei ATCC334 produces siderophores in cancer environments, inhibiting colorectal tumor progression by activating the JNK signaling pathway. Furthermore, Kita et al.^[^ ^37^ ^]^ showed that these siderophores induce DNA fragmentation and poly(ADP‐ribose) polymerase cleavage via p53 activation, which promotes pancreatic cancer cell apoptosis. These findings open new avenues for using probiotics in cancer treatment.

##### Clinical Benefits

Specific probiotics have been found to improve symptoms in various diseases. For instance, *Bifidobacterium longum* NCC3001 has been shown to alleviate depression and gastrointestinal symptoms in severe IBS patients by reducing brain reactivity and lowering depression scores.^[^ ^38^ ^]^ Additionally, *Bifidobacterium longum* has demonstrated positive effects beyond psychological health, such as promoting differentiation of normal human epidermal keratinocytes (NHEKs) and upregulating regenerative markers, which is crucial in regulating skin immune responses and preventing eczema.^[^ ^39^ ^]^

FMT has been shown to increase the relative abundance of Lactobacillus species, thereby promoting gut health by restoring microbial diversity and enhancing immune functions.^[^ ^40^ ^]^

#### 2.1.2 Role and Function of Pathogenic Microorganisms

Pathogens are microorganisms that can cause diseases by disrupting the host's immune system, causing inflammation, and compromising gut barrier function. Overgrowth of pathogenic bacteria in the gut can lead to chronic inflammation and various gastrointestinal disorders.

##### Immune Evasion via Biofilm Formation

Pathogens evade immune detection by forming biofilms, structured multicellular communities enveloped in a self‐produced extracellular matrix. This biofilm impedes the diffusion of antibiotics, facilitates the spread of resistance genes, and helps pathogens evade the host's immune defenses, contributing to disease progression.^[^ ^41^ ^]^ Pathogenic bacteria, such as *C. difficile* and *E. coli*, form biofilms that enhance their virulence and immune escape, thus exacerbating gut diseases.^[^ ^29^ ^]^

##### Pro‐Inflammatory Effects

Pathogens stimulate immune cells and trigger the release of inflammatory cytokines (such as TNF‐α, IL‐6), which causes chronic inflammation. For instance, *Clostridioides difficile* and *Escherichia coli* overgrowth results in gut barrier damage and abnormal immune activation, which contributes to the development of persistent inflammation.^[^ ^29^, ^42^ ^]^

##### Gut Permeability and Translocation Injury

Pathogens damage the intestinal mucosa by destroying mucus and consuming goblet cells, which leads to increased intestinal permeability. This allows pathogens to translocate from the gut into other parts of the body, causing systemic infections and worsening disease pathology.^[^ ^42^, ^43^ ^]^

FMT has been shown to reduce pathogenic bacteria and their biofilm formation, thereby alleviating the inflammatory responses associated with pathogenic colonization in the gut.^[^ ^44^ ^]^

#### 2.1.3 Role and Function of Biofilms

Biofilms are microbial communities embedded in a self‐produced extracellular matrix that protect pathogens from external threats, including antibiotic treatment and immune system responses. Biofilm formation plays a crucial role in the persistence of infections and the progression of diseases.

##### Antibiotic Resistance

Biofilms act as a physical barrier that limits the diffusion of antibiotics, making bacteria within biofilms highly resistant to antibiotic treatment. This contributes to the emergence and spread of antibiotic‐resistant infections.^[^ ^41^ ^]^

##### Immune Evasion

Biofilms also prevent immune cells from effectively attacking pathogens. The dense extracellular matrix of biofilms hinders immune cell phagocytosis, while the accumulation of extracellular polysaccharides enhances the pathogen's adhesive ability, further helping it evade immune detection.^[^ ^45^ ^]^

##### Disease Progression

The formation of biofilms is closely linked to the progression of various diseases, including *colorectal cancer* (CRC) and *inflammatory bowel disease* (IBD). The presence of mature biofilms in infected tissues can serve as an indicator of early disruptions, damage, and pathogenic infections in the intestinal environment.^[^ ^46^ ^]^

FMT has been demonstrated to break down biofilms formed by pathogenic bacteria, thereby reducing their virulence and allowing the immune system to function more effectively in eliminating these pathogens.^[^ ^44^ ^]^

#### 2.1.4 FMT's Role in Modulating Microbial Populations

FMT has a profound impact on the regulation of gut microbial populations, including probiotics, pathogens, and biofilms:

##### Restoring Probiotics

FMT promotes the growth of beneficial bacteria like *Lactobacillus* and *Bifidobacterium*, restoring microbial diversity and boosting the immune system. These beneficial bacteria enhance gut health by improving gut barrier function, suppressing pathogenic growth, and modulating immune responses.^[^ ^40^ ^]^

##### Suppressing Pathogens

FMT has been shown to reduce the abundance of pathogenic bacteria such as *C. difficile* and *E. coli*, leading to a reduction in gut inflammation and restoration of normal gut function.^[^ ^44^ ^]^

##### Disrupting Biofilms

FMT disrupts biofilms formed by pathogenic bacteria, which not only improves immune responses but also enhances the effectiveness of antibiotics and other treatments in eliminating infections.^[^ ^44^ ^]^

### 2.2 Metabolites and Their Derivatives

#### 2.2.1 Fatty Acids

According to their differing carbon chain lengths, fatty acids can be categorized as follows: short‐chain fatty acids, with a carbon chain containing fewer than 6 carbon atoms, also known as volatile fatty acids; medium‐chain fatty acids, with a carbon chain containing 6–12 carbon atoms, primarily composed of caprylic acid (C8) and capric acid (C10); and long‐chain fatty acids, with a carbon chain containing more than 12 carbon atoms. Literature reports that the fatty acids influencing the gut microbiota are primarily represented by short‐chain fatty acids (SCFAs) and long‐chain fatty acids (LCFAs).^[^ ^47^, ^48^ ^]^

The microbial fermentation of dietary carbohydrates primarily leads to the formation of short‐chain fatty acids (SCFAs), with acetic acid (C2), propionic acid (C3), and butyric acid (C4) being the beneficial SCFAs studied in clinical research. Following absorption in the intestines, SCFAs are distributed and metabolized differently within host cells. Acetate can exist in peripheral blood at higher concentrations, serving as a nutrient for cellular metabolism and playing roles in appetite regulation, energy expenditure, and immune responses.^[^ ^30^ ^]^ Propionate is a major substrate for hepatic gluconeogenesis and has similar effects to statin drugs, inhibiting the cholesterol synthesis pathway. Butyrate provides energy, supports the renewal of intestinal epithelium, and also exhibits immunosuppressive functions.

The role of SCFAs in modulating immune responses and disease progression has garnered extensive attention. By enhancing the number of immune cells and activating the expression of anti‐inflammatory factors, SCFAs contribute significantly to controlling disease development. Recent studies have demonstrated that SCFAs exert crucial immunoregulatory effects through interaction with their specific receptors, such as GPR41, GPR43, and GPR109A.^[^ ^49^ ^]^ These receptors are predominantly expressed on immune cells, where they play pivotal roles in regulating immune responses.

The importance of SCFA receptors in immune modulation can be elaborated from several perspectives: First, the widespread expression of GPR43 on immune cells facilitates the activation of immune cells and enhances their anti‐inflammatory functions. For instance, the activation of GPR43 promotes the proliferation and function of Tregs, which suppress excessive immune responses by secreting anti‐inflammatory cytokines, such as IL‐10, thereby maintaining immune tolerance and attenuating chronic inflammation.^[^ ^50^ ^]^ Second, SCFAs, via their receptors—particularly GPR109A—play a crucial role in the gut immune system. GPR109A, expressed on intestinal epithelial cells, helps inhibit excessive inflammatory responses, thereby preserving gut barrier function and preventing harmful substances from triggering an overactive immune reaction.^[^ ^51^ ^]^ Furthermore, the activation of GPR41 and GPR43 strengthens the intestinal immune barrier, modulating immune responses to enable the immune system to appropriately respond to external threats, such as bacteria and viruses, while avoiding attacks on host tissues. This mechanism is especially important in diseases like CRC, where an excessive immune response can promote tumor development and progression.

Additionally, the influence of SCFA receptors extends beyond local intestinal immune modulation and may indirectly impact systemic immune status through the regulation of gut microbiota and intestinal barrier function. For example, a study by Kobayashi et al.^[^ ^52^ ^]^ demonstrated that GPR41 and GPR43, through their regulation of p1 and JNK signaling pathways in renal epithelial cells, inhibit the expression of MCP‐38 induced by TNF‐α, thereby preventing kidney inflammation and fibrosis. Moreover, research suggests that GPR109A's interaction with butyrate may exert anti‐inflammatory effects.^[^ ^53^, ^54^ ^]^ Butyrate plays a multifaceted and complex role in the prevention and treatment of colorectal cancer. These mechanisms include promoting cell differentiation and inhibiting cancer cell proliferation through GPR43 activation in intestinal epithelial cells^[^ ^55^, ^56^ ^]^; inducing epigenetic modifications, such as inhibiting histone deacetylase (HDAC) activity, which leads to increased histone acetylation and activation of genes associated with cell cycle regulation and apoptosis^[^ ^57^ ^]^; modulating immune responses by enhancing intestinal epithelial barrier function, reducing chronic intestinal inflammation, and inhibiting immune reactions related to colorectal cancer^[^ ^58^ ^]^; and suppressing cell proliferation by regulating cell cycle proteins, inhibiting anti‐apoptotic factors, and activating apoptotic pathways, effectively inhibiting cancer cell proliferation and promoting their natural apoptosis.^[^ ^59^, ^60^ ^]^ These combined mechanisms collectively reduce cancer cell growth and metastasis.^[^ ^61^, ^62^ ^]^

SCFAs play a critical role in modulating IBD and CRC. Animal studies have shown that in a model of ulcerative colitis (UC), *Gpr43^‐/‐* mice, when treated with dextran sulfate sodium (DSS), exhibit more severe colonic inflammation compared to wild‐type (WT) mice, indicating a protective role of Gpr43 in regulating colonic inflammation.^[^ ^63^ ^]^ Furthermore, research has shown that acetate effectively alleviates DSS‐induced colonic inflammation, with this anti‐inflammatory effect significantly reduced in *Gpr43^‐/‐* mice, further emphasizing the central role of Gpr43 in SCFA‐mediated anti‐inflammatory responses.^[^ ^64^ ^]^ In clinical research, butyrate has been widely used to alleviate colonic inflammation due to its ability to enhance intestinal epithelial barrier function and regulate immune responses.^[^ ^65^ ^]^ Clinical trial results indicate that butyrate, administered via enema, significantly improves the condition of UC patients, primarily by acting locally on the colonic mucosa to reduce inflammation and promote healing.^[^ ^66^ ^]^

In colorectal cancer research, the *ApcMin/+* mouse model showed that *Gpr109A^–/–* mice form more colonic polyps than control mice, indicating that GPR109A acts as a tumor suppressor in the prevention of colorectal cancer.^[^ ^67^ ^]^ In a model of colon cancer induced by azoxymethane (AOM) and DSS, *Gpr109A^−/−* mice also developed more colonic polyps than the control group, further supporting the essential role of GPR109A in colorectal cancer prevention.^[^ ^64^ ^]^ Notably, in colorectal cancer patients, the expression of GPR109A is significantly reduced in tumor tissues, while it is higher in normal colonic mucosa, suggesting that GPR109A may serve as a potential suppressor of colorectal cancer.^[^ ^68^ ^]^ Additionally, studies have shown that nicotinic acid, a GPR109A agonist, induces apoptosis in colorectal cancer cells, suggesting that GPR109A agonists could become novel therapeutic agents for colorectal cancer.^[^ ^69^ ^]^

Long‐chain fatty acids (LCFAs) must be obtained from dietary sources, and an increase in saturated LCFAs has been positively correlated with the abundance of Prevotella, *Lactobacillus*, and *Bifidobacterium genera*.^[^ ^70^ ^]^ Research on LCFAs has mainly focused on macrophages, but the quantity and type of LCFAs in the environment may disrupt the function of most immune cells. GPR120 is a known metabolite of LCFAs, and studies have shown that activating GPR120 in macrophages can inhibit the activation of the NLRP3 inflammasome,^[^ ^71^ ^]^ reduce the maturation and secretion of pro‐inflammatory cytokines IL‐1β and IL‐18, and suppress intestinal inflammatory responses.^[^ ^72^ ^]^

After FMT treatment, while the overall FA levels remained unchanged, a significant shift was observed in the ratio between inflammatory and non‐inflammatory FAs, with a decrease in the proportion of inflammatory FAs, possibly linked to a reduction in inflammation levels post‐treatment. Furthermore, the fecal samples of the subjects exhibited a marked increase in microbial diversity, indicating an improvement in the microbial community structure. These alterations led to a microbiota composition of the subjects closer to that of healthy donor characteristics, which may serve as a crucial indicator of the effectiveness of FMT.^[^ ^73^ ^]^ FMT may influence SCFA metabolism by correcting intestinal dysbiosis or directly transferring SCFAs, which could underlie the effects observed in IBD (refer to the following section).

#### 2.2.2 Protein Enzymes

Not only do the microorganisms in the gut influence health, but the protein enzymes secreted by these microorganisms play a crucial role in the development of diseases. AimA is an immunoregulatory protein secreted by *Pseudomonas aeruginosa*,^[^ ^31^ ^]^ which can inhibit the growth of harmful bacteria in the host, reduce intestinal inflammation, prevent excessive accumulation of neutrophils, thereby avoiding septic shock. Some bacteria also secrete amino acid‐derived antibiotics to combat diseases. For example, the intestinal bacteria *Clostridium scindens* and *Clostridium sp*. each secrete the tryptophan‐derived antibiotics 1‐acetyl‐b‐carotine and turbomycin A,^[^ ^74^ ^]^ these two antibiotics inhibit bacterial growth by blocking the formation of septa during bacterial division, thereby suppressing the growth of C. difficile and other intestinal bacteria.

#### 2.2.3 Toxic Compounds

Aromatic amino acids produce a series of metabolites after bacterial fermentation, some of which are toxic, including certain nitrogen‐containing compounds, ammonia, amines, and sulfides. Some nitrogen‐containing compounds, especially nitrites, induce significant DNA damage, including DNA double‐strand breaks, increasing the risk of cancer.^[^ ^32^ ^]^ In addition, Windey et al.^[^ ^75^ ^]^ have shown that low concentrations of ammonia are also carcinogenic, as demonstrated in animal models to be associated with mucosal damage and colorectal adenocarcinoma. The discovery of polyamine synthesis in intestinal bacteria has revealed the toxicity of high levels of polyamines, contributing to oxidative stress and various diseases, including cancer. Olin‐Sandoval et al.^[^ ^76^ ^]^ demonstrated that oxidative stress induced by polyamine breakdown metabolism is its toxic mechanism. Di Martino et al.^[^ ^77^ ^]^ found that *Streptococcus pneumoniae*, *Helicobacter pylori*, and *Salmonella enterica Typhi* utilize polyamines to optimize their adaptability within the host and enhance their own virulence. Under anaerobic conditions, sulfate‐reducing bacteria (such as *Desulfovibrio*) reduce sulfate to H2S, which is not only toxic to colon cells but also inhibits butyrate oxidation, disrupting the integrity of the colonic cell barrier, promoting the development of inflammatory bowel disease, or at least increasing the risk of recurrence.^[^ ^78^ ^]^ Furthermore, Blachier et al.^[^ ^79^ ^]^ observed that at very low concentrations, sulfides induce DNA damage through the generation of reactive oxygen species, further promoting the development of cancer.^[^ ^80^ ^]^ FMT can reduce the production of unnecessary toxic compounds by modulating the intestinal microbiota.

#### 2.2.4 Bile Acids

Bile acids (BAs), cholesterol derivatives, play crucial roles in lipid metabolism, antimicrobial defense, and glucose homeostasis. Stored in the gallbladder, BAs are released into the small intestine after food intake, where they facilitate fat emulsification and nutrient absorption. Subsequently, BAs are reabsorbed by the liver, completing their enterohepatic circulation.^[^ ^81^, ^82^ ^]^ The interaction between the gut microbiota and host bile acid metabolism is vital for maintaining intestinal homeostasis and overall health. The gut microbiota finely regulates bile acid metabolic pathways, involving a series of complex biochemical reactions that profoundly impact the chemical structure and biological functions of bile acids.^[^ ^82^ ^]^ In this process, primary bile acids undergo microbial‐mediated transformations, yielding structurally diverse secondary bile acids. Key enzymatic reactions involved in this transformation include hydrolysis, dehydroxylation, oxidation, isomerization, esterification, and desulfurization.^[^ ^83^, ^84^ ^]^ Specific gut bacteria, such as *Bacteroides*, *Clostridium*, *Lactobacillus*, *Bifidobacterium*, *Enterococcus*, *Ruminococcus*, and *Listeria*, play pivotal roles in bile acid hydrolysis through bile salt hydrolase (BSH) activity.^[^ ^82^, ^84^, ^85^, ^86^ ^]^ Additionally, hydroxysteroid dehydrogenases produced by the microbiota are crucial for bile acid oxidation, isomerization, and dehydroxylation.^[^ ^87^ ^]^ *Clostridium* and *Bacteroides* enhance bile acid dehydroxylation,^[^ ^88^ ^]^ while *Bacteroides*, *Firmicutes*, *Clostridium*, *Escherichia*, *Eggerthella*, *Ruminococcus*, *Streptococcus*, and *Lactobacillus* play synergistic roles in bile acid oxidation and isomerization.^[^ ^87^, ^88^ ^]^ Esterification reactions are primarily facilitated by *Bacteroides*, *Firmicutes*, and *Lactobacillus*,^[^ ^89^ ^]^ whereas *Clostridium*, *Bacteroides*, *Peptostreptococcus*, and *Pseudomonas* play essential roles in bile acid desulfurization.^[^ ^85^, ^88^ ^]^

The role of the gut microbiota in bile acid metabolism extends beyond the conversion of primary bile acids to secondary bile acids; it also indirectly influences bile acid biosynthesis by finely regulating the expression of bile acid synthesis enzymes. Specifically, Say et al.^[^ ^90^ ^]^ showed that the gut microbiota downregulates taurine‐conjugated bile acid levels in the intestine while upregulating cholesterol 7α‐hydroxylase (CYP7A1) expression in the liver, without affecting cholesterol‐7α‐hydroxylase activity, thereby modulating bile acid composition. This finding underscores the complex interplay between the gut microbiota and host metabolism. Furthermore, Kwon et al. reported that in mice fed *Lactobacillus plantarum*, the expression of bile acid synthesis‐related genes such as Cyp7a1, 7α‐hydroxylase, Cyp27a1, and Cyp8b1 in the liver was significantly elevated, enhancing bile acid synthesis capacity.^[^ ^91^ ^]^ These findings suggest that certain probiotics can activate the host's bile acid synthesis pathways, potentially benefiting metabolic health.

Bile acids exert multifaceted effects on the gut microbiota, not only modulating microbial composition but also influencing its functional capacities. As antimicrobial molecules, bile acids directly interact with bacterial membranes, disrupting membrane integrity, inducing DNA damage, and causing protein denaturation, which inhibits the growth and proliferation of certain bacteria.^[^ ^92^ ^]^ For example, unconjugated bile acids generally exhibit stronger antimicrobial activity than conjugated bile acids,^[^ ^87^, ^93^ ^]^ with cholic acid (CA) inhibiting the growth of Gram‐negative bacteria and increasing the abundance of bacteria with bile acid 7α‐dehydroxylase activity, such as *Clostridium XIVa*.^[^ ^94^, ^95^ ^]^ Deoxycholic acid (DCA) primarily inhibits the growth of Gram‐positive bacteria, such as *Clostridium perfringens* and *Bacteroides fragilis*, by disrupting bacterial membrane integrity.^[^ ^96^, ^97^, ^98^ ^]^ Chronic supplementation with exogenous DCA can deplete BSH‐containing bacteria and induce intestinal inflammation in mice, thereby disrupting bile acid metabolic balance.^[^ ^99^ ^]^ On the other hand, ursodeoxycholic acid (UDCA) can improve gut microbiota imbalances by modulating the ratio of *Firmicutes* to *Bacteroidetes*, promoting ulcer epithelial healing, and exhibiting anti‐inflammatory, anti‐apoptotic, and antioxidant effects in mouse intestines.^[^ ^100^, ^101^ ^]^ IsoalloLCA inhibits the growth of Gram‐positive bacteria, such as *Clostridioides difficile*, while having no effect on Gram‐negative bacteria.^[^ ^102^ ^]^ Additionally, the concentration of bile acids influences the growth of different bacteria in the gut: high concentrations favor the growth of bacteria with 7α‐dehydroxylase activity, while low concentrations promote the growth of Gram‐negative bacteria.^[^ ^103^ ^]^ Thus, bile acids finely regulate the gut microbiota, maintaining intestinal microbial homeostasis and playing a critical role in host health.

Recent studies suggest that correcting bile acid metabolism may be a key mechanism for FMT in curing and preventing recurrent CDI.^[^ ^104^ ^]^ Latest advances indicate that certain bile acids, such as taurocholic acid (TCA), promote spore germination of *Clostridioides difficile*, while secondary bile acids, like deoxycholic acid (DCA), effectively inhibit its vegetative growth and toxin activity.^[^ ^105^, ^106^ ^]^ In the human gut, the conversion of primary bile acids to secondary bile acids is mediated by enzymes produced by the gut microbiota, with bile salt hydrolase (BSH) and 7‐α‐dehydroxylase playing crucial roles.^[^ ^107^ ^]^ In recurrent CDI (rCDI) patients, these key microbial enzymes are often deficient, leading to the accumulation of TCA and reduction of DCA, which may be a major factor in the persistence of *C. difficile* infection.^[^ ^108^, ^109^ ^]^ FMT restores these key microbial populations, adjusts the bile acid environment, reduces TCA levels, and increases DCA levels, effectively inhibiting the growth and toxin activity of *C. difficile*.^[^ ^110^, ^111^ ^]^ After FMT, circulating fibroblast growth factor (FGF)‐19 levels increase, consistent with the activation of the FXR‐FGF pathway, which may help alleviate inflammation and enhance intestinal barrier integrity.^[^ ^112^ ^]^ FXR activation may promote the recovery from colitis, reduce colonic inflammation, and improve intestinal barrier function.^[^ ^110^ ^]^ In conclusion, FMT restores bile acid metabolism, particularly BSH function, and activates the FXR‐FGF signaling pathway, providing therapeutic effects against rCDI. This not only directly impacts *C. difficile*, but also modulates host metabolism and immune responses, highlighting the potential mechanisms by which FMT regulates bile acid metabolism and host immune responses in the treatment of rCDI.^[^ ^113^ ^]^ These findings offer new perspectives on the therapeutic potential of FMT in managing recurrent CDI.

## 3 The Application of Fecal Microbiota Transplantation in Gastrointestinal Disorders

After the disruption of gut microbiota balance, alterations in the normal distribution and composition of gut microbiota, as well as the metabolites produced by the microbiota, lead to the occurrence and development of intestinal diseases. With the significant efficacy of FMT in the treatment of certain diseases being demonstrated, a multitude of animal model experiments have been conducted to explore its mechanism. The summarized mechanisms of FMT can be categorized into several aspects. First, FMT can accomplish the reconstruction of gut microbiota, introducing diverse beneficial bacteria to patients. Second, these beneficial bacteria and their metabolites exhibit antibacterial effects against harmful bacteria, aiding in the suppression of pathogen growth. Furthermore, FMT can promote immune regulatory responses by influencing the spectrum of gut microbial metabolites, indirectly affecting energy metabolism, lipid metabolism, hormone regulation, and thereby ameliorating the pathological and physiological processes of intestinal diseases. Currently, FMT has been widely attempted in the treatment of intestinal diseases such as Clostridioides difficile infection, inflammatory bowel disease, constipation, short bowel syndrome, irritable bowel syndrome, colorectal tumors, among others, and has achieved favorable clinical outcomes (**Table** **2**).

**Table 2:** Possible mechanisms of disease in each intestinal disorder and the mechanisms and effects of FMT treatment. We will present the aforementioned content in a more intuitive **table format**, using “↑” to represent an **increase** and “↓” to represent a **decrease** in the quantities of corresponding microbial populations, cells, cellular products, or other substances.

	Pathogenic mechanism				FMT		
	The gut flora		Metabolites and their derivatives		Mechanism		Effectiveness (cure/remission rate)
CDI	*C.difficile*↑	[114, 115]	Collagenase, Haluronidase, Chondroitin sulfatase Benterotoxin A toxin, Bcytotoxin B toxin	[116]	Correct bile acid metabolism correct the gut flora	[110, 111, 117, 118]	53–93% ^[^ ^119^, ^120^ ^]^
IBD	*Bacteroidetes*↓, *Firmicutes*↓	[121]	Oxidative stress Produce inflammatory cytokines	[122, 123, 124, 125, 126]	Correct intestinal flora disorders Produce butyrate Improve oxidative stress produce immunoglobulins (IgA, IgG)	[127, 128, 129, 130, 131, 132, 133]	52–78% ^[^ ^19^, ^20^ ^]^
Constipation	*Lactobacillus*↓, *Clostridium*↓	[134]	SERT↑, 5‐HT↓	[135]	Regulate protein digestion and absorption pathways	[136]	73.5%^[^ ^137^ ^]^
SBS	*clostridium firmicutes*↓	[138]	SCFAs↓	[138]	Firmicutes, SCFAs pro‐inflammatory bacteria↓	[139]	–
IBS	*Bifidobacteria*, *Enterococcus faecalis*↓ *E.coli*, *Bacillus were* ↑	[140]	–		Regulat microbiome‐Gut‐brain Axis(MGBA) Regulate GABA receptors and reduce corticosterone	[141, 142, 143]	70%^[^ ^144^ ^]^
CRC	*Bifidobacteria*, *Lactobacillus*, *Bacteroides*↓ *E.coli*, *Bacteroides fragilis*, *Clostridium nuclei*↑	[145]	IL‐6, TNF‐α↑ Produces extracellular superoxide and hydrogen peroxide Produce ammonia, phenols, hydrogen sulfide, NOC	[75, 146, 147, 148, 149]	Correct intestinal flora disorders CD8+ T Cell, CD49bNK Cell, Foxp3 Treg cells↓ As radiological protection agent	[150, 151]	–

### 3.1 Infection of *Clostridioides difficile* in the Gastrointestinal Tract

Clostridioides difficile(*C. difficile*), a Gram‐positive, spore‐forming anaerobic bacillus, is widely distributed in the intestinal tracts of both humans and animals.^[^ ^114^, ^115^ ^]^ The clinical manifestations of infected individuals vary, ranging from asymptomatic carriage to varying degrees of diarrhea, and ultimately to the most severe life‐threatening colitis that can lead to death.^[^ ^115^, ^152^ ^]^ Over the past decade, the frequency and severity of CDI(Clostridioides difficile infection) have been continuously increasing worldwide, making it one of the most common hospital‐acquired infections.^[^ ^115^ ^]^

Compared to healthy individuals, CDI patients exhibit a significant reduction in the abundance of *Bacteroidetes* and SCFA‐producing bacteria, such as *Firmicutes*, *Roseburia*, and *Ruminococcus*, as well as *Bacteroidetes* species, including *Bacteroides*, *Prevotella*, and *Alistipe*s.^[^ ^153^ ^]^ Specifically, SCFA‐producing bacteria play a crucial role in maintaining gut microbial balance by enhancing intestinal barrier function and mucosal immunity.^[^ ^154^, ^155^ ^]^ However, in CDI development, the reduced abundance of these bacteria impairs the ability to effectively suppress *C. difficile* proliferation.^[^ ^156^ ^]^ Furthermore, the loss of microbial diversity and the depletion of specific bacterial populations may disrupt fatty acid and bile acid metabolism, further promoting *C. difficile* growth and contributing to CDI progression.^[^ ^102^, ^108^ ^]^ The use of antibiotics may also disrupt the gut microbiota balance, weakening its ability to suppress pathogens, thereby increasing the abundance of opportunistic pathogens and creating a favorable environment for their colonization.^[^ ^157^ ^]^ Additionally, *C. difficile* infects humans via spores and, being non‐invasive, produces toxic compounds such as collagenase, hyaluronidase, chondroitin‐sulfatase, enterotoxin A, and cytotoxin B, which damage the epithelial cytoskeleton, induce neutrophil adhesion, and trigger local inflammatory responses, ultimately compromising the integrity and function of the intestinal barrier.^[^ ^116^ ^]^

rCDI (recurrent CDI) treatment primarily relies on the use of antibiotics, including metronidazole, vancomycin, and fidaxomicin.^[^ ^158^ ^]^ However, the cure rate is relatively low (20–30%), possibly due to the emergence of resistance with antibiotic use, which further disrupts the stability of the gut microbiota, weakening or losing the resistance to *C. difficile*, leading to colonization and growth of *C. difficile*, ultimately resulting in recurrent infections.^[^ ^159^ ^]^ On the other hand, using FMT to treat rCDI is a more effective and cost‐effective method for preventing recurrence.^[^ ^160^ ^]^ From 2011 to 2021, over 10 000 CDI patients globally have benefited from FMT.^[^ ^21^ ^]^ The cure rate of using FMT for recurrent or refractory CDI ranges from 68% to 100%, with a cure rate of 53% to 93% for a single FMT treatment.^[^ ^119^, ^120^ ^]^ Minkoff et al.,^[^ ^161^ ^]^ who included 320 studies, concluded that in immunocompetent adults with recurrent CDI, FMT can significantly reduce the recurrence rate of CDI compared to alternative therapies such as antibiotics. Although FMT has shown promising results in treating CDI, its precise mechanisms of action remain incompletely understood. It is currently believed that FMT may exert its therapeutic effects through competitive inhibition of *C. difficile* growth, primarily by providing microbial populations that have a nutritional advantage and by creating an environment that is unfavorable for *C. difficile* survival.^[^ ^162^ ^]^ Metagenomic analysis has revealed that, compared to healthy individuals, CDI patients exhibit a decrease in gut microbiota richness and diversity, along with an increase in *Proteobacteria* species, while the levels of *Firmicutes* and *Bacteroidetes* species are reduced.^[^ ^163^ ^]^ FMT restores gut health and suppresses *C. difficile* growth by re‐establishing microbial balance, specifically by increasing *Firmicutes* and *Bacteroidetes* species while decreasing *Proteobacteria* species, which is considered a key mechanism underlying its therapeutic success.^[^ ^163^ ^]^

The efficacy of FMT also involves modulation of bile acid metabolism, which is crucial for controlling *C. difficile* spore germination. Primary bile acids promote spore germination, whereas secondary bile acids (e.g., lithocholic acid salts) inhibit this process. Certain gut microbes, particularly those from the *Lachnospiraceae* and *Ruminococcaceae* families, possess the ability to convert primary bile acids into secondary bile acids, a transformation that is critical for suppressing *C. difficile*.^[^ ^82^, ^105^ ^]^ The changes in bile acid composition observed in fecal samples before and after FMT suggest that FMT can correct the bile acid imbalance in patients with rCDI, restoring it to a healthier state.^[^ ^111^ ^]^Sialic acid metabolism is another key pathway that FMT may influence. Ng et al. demonstrated in a mouse model that antibiotic‐induced disruption of the endogenous microbiota led to an increase in free mucosal sialic acid, which provided an energy source for *C. difficile*, thereby promoting its expansion in the gut.^[^ ^164^ ^]^ FMT may counter this effect by enhancing the utilization of sialic acid by symbiotic bacteria, thereby depriving *C. difficile* of this energy source and contributing to therapeutic outcomes. Beyond these mechanisms, FMT may also exert effects through other yet‐to‐be‐fully elucidated pathways, such as activating protease activity to neutralize *C. difficile* toxins, releasing short‐chain fatty acids and other small molecules to enhance host cell defenses, and directly inhibiting *C. difficile* activity via bacteriocin‐like mechanisms.^[^ ^165^ ^]^

Currently, FMT has been recommended as the standard approach for treating recurrent refractory CDI by multiple expert guidelines. Nonetheless, there remains controversy surrounding the optimal timing of FMT implementation. While it is generally advised to consider FMT after the third recurrence of CDI,^[^ ^158^, ^166^, ^167^ ^]^ studies have indicated that FMT may also be beneficial for patients experiencing second or even first recurrences, depending mainly on the clinical condition of the patient and the presence of severe complications. In certain scenarios, early consideration of FMT may be warranted, such as in cases where the patient has a significant medical history, requires prolonged hospitalization or long‐term care, or when urgent treatment is needed for complications. The potential benefits of FMT for patients experiencing a first episode of CDI are still not clearly defined. However, in cases of refractory or severe CDI during the initial episode, FMT has been shown to be effective and may even be life‐saving.^[^ ^168^ ^]^

### 3.2 Recurrent and Refractory Inflammatory Bowel Disease

IBD is an idiopathic inflammatory disorder affecting the ileum, rectum, and colon. It includes conditions such as UC and CD. The main treatment strategies for IBD currently include pharmacological therapy, immune modulation, and surgical intervention in refractory cases. Commonly used drugs include anti‐inflammatory agents (e.g., corticosteroids and immunosuppressants), biologic therapies (e.g., anti‐TNF‐α antibodies and IL‐12/23 inhibitors), and topical medications (e.g., intestinal lubricants and antibiotics).^[^ ^170^, ^171^ ^]^ However, many patients have a poor response to these traditional therapies, especially those with recurrent or refractory cases. Immunosuppressants and biologics may be associated with serious side effects, and long‐term reliance on medications may lead to drug resistance or toxicity.^[^ ^172^, ^173^ ^]^ In recent years, FMT has garnered significant attention as an emerging therapeutic approach. Studies suggest that FMT may offer a novel alternative treatment for IBD patients, particularly those who do not respond well to conventional drug therapies.

It is widely believed that dysbiosis of the gut microbiota and its metabolic products is one of the factors leading to the development of IBD.^[^ ^174^ ^]^ This dysbiosis triggers abnormal immune responses in the gut, initiating mucosal inflammation and promoting IBD progression.^[^ ^126^ ^]^ Numerous studies have shown a close relationship between gut microbiota changes and IBD, with IBD patients typically exhibiting significantly reduced microbial diversity.^[^ ^175^ ^]^ In particular, the proportions of Firmicutes (especially Clostridia) and Bacteroidetes are markedly reduced, while these bacteria dominate the healthy human gut microbiota.^[^ ^121^ ^]^

FMT has been shown to significantly increase microbial diversity in patients with UC, particularly in those who experience clinical symptom relief. Specifically, the concentrations of *Hallii* and *Roseburia inulivorans* were found to increase, accompanied by higher levels of SCFAs.^[^ ^176^ ^]^ These results highlight the potential importance of FMT in adjusting gut microbiota balance and promoting health. In contrast, patients whose symptoms did not improve exhibited increased abundance of *Fusobacterium gonidiaformans*, *Sutterella wadsworthensis*, and *Escherichia* species, along with elevated levels of lipopolysaccharide (LPS). These changes in microbial composition and metabolic products may be linked to inflammation and impaired gut barrier function, influencing the effectiveness of FMT. Further research has shown that the microbial composition of donor feces is directly correlated with the efficacy of FMT. Specifically, the abundance of *Bacteroides* was positively correlated with successful FMT outcomes, while increased *Streptococcus* species were associated with poor treatment responses.^[^ ^176^ ^]^ This finding underscores the importance of selecting appropriate donors and considering the characteristics of their microbiota when conducting FMT treatment. In patients with long‐term remission, there was a significant increase in butyrate production and the abundance of butyrate‐producing bacteria.^[^ ^176^ ^]^ As an SCFA, butyrate is crucial for maintaining gut health and regulating immune responses. This result further confirms the effectiveness of FMT in adjusting the gut microbiota and promoting long‐term symptom relief in UC patients.

**Figure 4:**
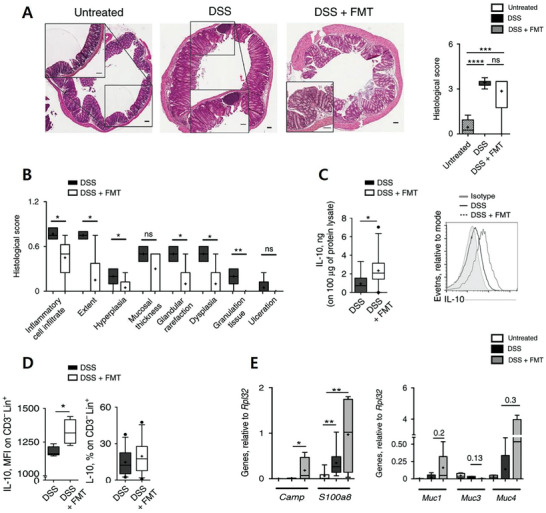
The diagram illustrates the effect of FMT on improving *dextran sulfate sodium* (DSS)‐induced colitis in mice. It evaluates the changes in histopathological scores, cytokine levels, immune cell function, and the expression of relevant genes. A). Histopathological Observation of Colon Tissue.The left panel shows hematoxylin and eosin (HE) staining of colon tissues from untreated, DSS‐induced, and DSS + FMT‐treated mice. In the DSS group, significant inflammation, mucosal destruction, and glandular atrophy were observed, while tissue damage was notably improved in the DSS + FMT group. The right panel displays histological scores, with the DSS + FMT group showing significantly lower scores compared to the DSS group (*p* < 0.001), indicating a protective effect of FMT on colon tissue damage. B). Sub‐item Analysis of Histological Scores. Various pathological indicators such as inflammation, epithelial injury, and crypt deformation were compared. FMT significantly reduced scores for inflammation, epithelial injury, and crypt destruction (*P < 0.05, ^*^ *p* < 0.01), while having no significant effect on granuloma formation and ulceration (ns). C). IL‐10 Expression in Colon Tissue. The left bar chart shows that IL‐10 levels were significantly higher in the DSS + FMT group (^*^ *p* < 0.05). The right flow cytometry data further corroborate that IL‐10 expression was enhanced in the DSS + FMT group, suggesting that FMT may exert its effect by modulating the anti‐inflammatory cytokine IL‐10.(D) IL‐10 Expression in T Cells.FMT treatment significantly increased IL‐10 expression in CD3+ T cells, as shown by the increase in mean fluorescence intensity (MFI) (*p* < 0.05) in the left panel, and by a higher percentage of IL‐10‐positive CD3+ T cells in the right panel. (E). Antimicrobial Peptide and Mucin Gene Expression Analysis.Antimicrobial peptide genes: FMT significantly upregulated the expression of Camp and S100a8 genes (^*^ *p* < 0.05, ^*^ *p* < 0.01), indicating that FMT may alleviate inflammation by enhancing antimicrobial barrier function.Mucin‐related genes: FMT had no significant effect on the expression of Muc1 and Muc3 genes, but showed a tendency to upregulate Muc4 expression, although this was not statistically significant. Reproduced with permission.^[^ ^169^ ^]^ Copyright 2018, Springer Nature.

Overall, FMT improves acute and chronic inflammation in IBD by correcting gut dysbiosis, regulating microbial metabolism, promoting anti‐inflammatory cytokines, inhibiting pro‐inflammatory cytokines, reducing oxidative stress, and facilitating mucosal barrier repair.^[^ ^127^, ^128^, ^129^, ^130^ ^]^ It helps maintain epithelial integrity, suppresses Th1 cell differentiation and T‐cell activity, and restores immune dysregulation by reducing leukocyte adhesion and inflammation.^[^ ^177^ ^]^ Research has shown that *Akkermansia muciniphila*, a beneficial bacterium involved in mucosal repair, is associated with the production of IL‐8, IL‐10, and SCFAs.^[^ ^178^, ^179^ ^]^ Kump et al.^[^ ^180^ ^]^ found that the relative abundance of *Akkermansia muciniphila* significantly increased in refractory UC patients after FMT. Burrello et al.^[^ ^169^ ^]^ demonstrated that FMT improved colonic inflammation in DSS‐induced experimental colitis mice, as indicated by reduced histological scores (**Figure** **4**A,B) and increased IL‐10 production from antigen‐presenting cells (Figure 4C,D). Furthermore, the antimicrobial peptides Camp and S100A8 were upregulated, and there was a tendency for increased expression of mucin genes Muc1 and Muc4 (Figure 4E). These mucins play an anti‐inflammatory role in pathogen response, suggesting that therapeutic FMT reduces colonic inflammation and restores gut homeostasis by activating multiple immune pathways. Moreover, FMT can alleviate oxidative stress,^[^ ^130^ ^]^ which is of significant importance in IBD pathogenesis. Reactive oxygen species overload can damage cytoskeletal proteins, disrupt tight junctions between intestinal epithelial cells, and increase epithelial permeability, ultimately leading to barrier dysfunction.^[^ ^132^ ^]^ FMT may also enhance the production of immunoglobulins such as IgA and IgG, which suppress immune inflammation.^[^ ^181^ ^]^

In recent years, preliminary studies on the use of FMT for the treatment of IBD have shown promising results. The research conducted by Kunde et al.^[^ ^19^ ^]^ has been particularly encouraging, with 78% of subjects experiencing clinical symptom relief within one week, and 67% maintaining relief after one month. Additionally, six refractor UC patients showed significant improvement in symptoms post‐FMT, with no evidence of UC recurrence during clinical follow‐ups ranging from 1 to 13 years without the use of any UC medication.^[^ ^182^ ^]^A recent systematic review reported that FMT could achieve clinical remission in 63% of IBD patients, and 76% of patients experienced sustained gastrointestinal symptom relief after discontinuing IBD‐related medications.^[^ ^183^ ^]^ A randomized controlled trial demonstrated that administering FMT via colonoscopy induced remission in active Ulcerative Colitis patients.^[^ ^184^ ^]^ However, in CD, there is a lack of randomized controlled trials and only small‐scale uncontrolled studies have been conducted, yielding mixed results. A meta‐analysis reported a composite remission rate of 52% among 71 CD patients who underwent FMT.^[^ ^20^ ^]^ Sokol et al.^[^ ^185^ ^]^ conducted a randomized controlled trial study and found that fecal microbiota transplantation can maintain the remission state of Crohn's disease.

Due to the limited observed effects and insufficient clinical trial data, FMT should still be considered an experimental approach for IBD. Future research may need to focus on selecting suitable donors (potentially utilizing super‐donors), identifying patients who are more likely to respond positively, and optimizing the anaerobic processing of donor fecal material. The timing of FMT in IBD patients remains uncertain: should it be used as an induction therapy or after initiation of induction therapy? Addressing these questions through research could pave the way for FMT to become a future therapeutic option for a subset of UC and CD patients.

The relationship between the inflammatory state and the success rate of FMT has been widely discussed. Studies have shown that IBD patients in active disease phases tend to have lower FMT success rates compared to those in remission.^[^ ^186^, ^187^ ^]^ The underlying reason is that active inflammation may disrupt the intestinal environment, potentially affecting the survival and colonization of the transplanted microbiota.^[^ ^188^ ^]^ Additionally, several studies have found a correlation between intestinal inflammation biomarkers, such as fecal calprotectin (FC), and clinical outcomes during FMT treatment.^[^ ^189^ ^]^ Specifically, elevated FC levels usually reflect active intestinal inflammation and may predict a poor response to FMT treatment.^[^ ^190^ ^]^ This suggests that inflammation plays a critical role in modulating the effectiveness of FMT, emphasizing the importance of controlling inflammation before or during FMT treatment to enhance its success. These findings further support the need for careful patient selection and monitoring of inflammatory biomarkers to optimize treatment strategies.

Regarding FMT therapy for IBD patients with recurrent rCDI, a meta‐analysis showed an initial cure rate of 80%. The initial cure rate for UC patients with CDI was higher than for CD patients (85% vs 79%), indicating the efficacy of FMT in treating rCDI in both CD and UC patients.^[^ ^191^ ^]^ Adverse events reported included IBD flares, with ongoing discussions regarding whether these flares are a result of FMT or CDI.

### 3.3 Constipation

Chronic functional constipation is a common gastrointestinal disease characterized by a reduction in defecation frequency, along with difficulty in defecation and dry stool. This condition not only has a significant impact on physiological functions but also can lead to varying degrees of psychological obstacles, severely affecting the quality of life. A recent study based on 16S rRNA microbial community analysis has shown that intestinal microbial dysbiosis exists in chronic constipation (CC),^[^ ^193^ ^]^ with constipation subtypes related to intestinal flora including slow transit constipation (STC) and chronic constipation with abdominal pain, also known as irritable bowel syndrome with constipation (IBS‐C: Rome II‐IV category).^[^ ^193^, ^194^ ^]^

**Figure 5:**
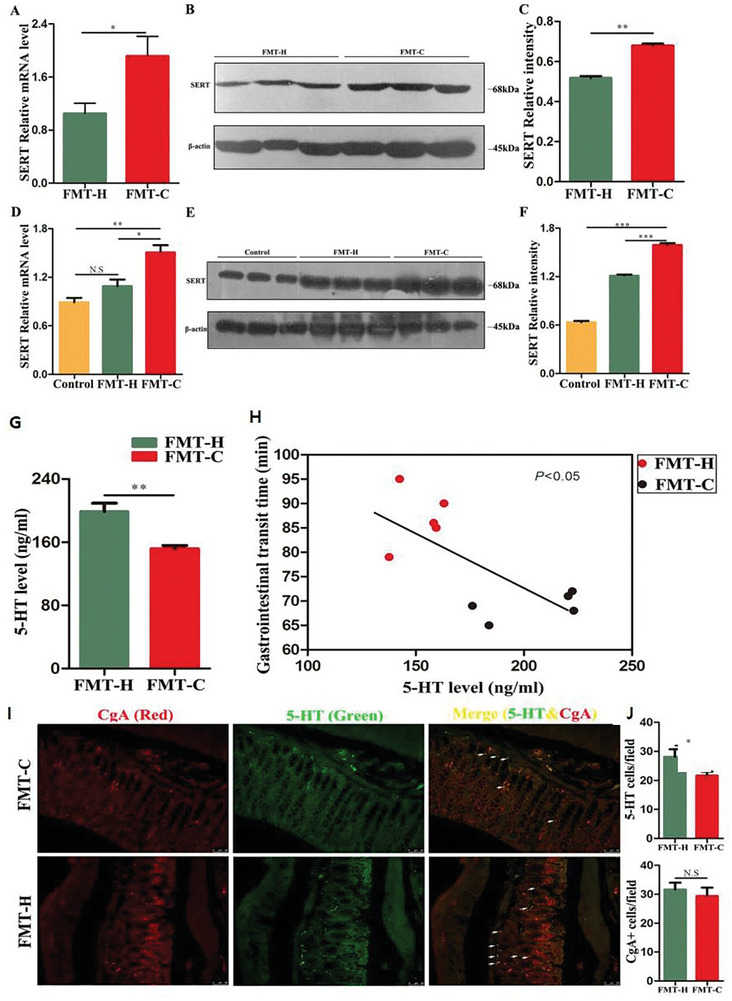
The fecal microbiota of constipated patients up‐regulated the SERT levels in the intestinal tissues of mice.A). The real‐time PCR findings demonstrated an upregulation of SERT mRNA levels in the intestinal tissues of the FMT‐C group, in contrast to the FMT‐H group. B). The intestinal protein levels of SERT in both groups were assessed through Western blot analysis, employing β‐actin as the internal control protein for overall protein quantification. C). Proteins were quantified via densitometry utilizing an Imaging processor program (Image J). D). In the same vein, the levels of SERT mRNA in Caco‐2 cells were observed following exposure to fecal microbiota from the three groups (blank control, FMT‐H, FMT‐C) for a duration of 3 h, with the fecal microbiota concentration ranging from 1 to 2000. E). The protein expression of SERT in Caco‐2 cells following treatment with fecal microbiota from the three groups for 3 h was assessed using Western blot analysis. F) The protein expression levels of SERT in Caco‐2 cells were quantitatively analyzed through densitometry using Image J. The FMT‐C group, which received the fecal microbiota from patients with constipation. FMT‐H group, the group that received the fecal microbiota of healthy controls; SERT, serotonin transporter; N.S, no significance; ^*^ *p* < 0.05, ^**^ *p* < 0.01, ^***^ *p* < 0.001. Constipated individuals have a decreased fecal microbiota, which led to lowered 5‐HT levels in the mouse intestinal tissues. G) The ELISA analysis revealed a reduction in the levels of 5‐hydroxytryptamine (5‐HT) in the FMT‐C group compared to the FMT‐H group. Additionally, the level of 5‐HT exhibited a significant correlation with GITT. I) 5‐hydroxytryptamine, also known as serotonin, is secreted by intestinal chromaffin cells. The protein Chromogranin A (CgA) is present in secretory granules of chromaffin cells. Immunofluorescence staining with primary antibodies against CgA was performed on paraffin sections of colonic tissues to label chromaffin cells (Red) and 5‐HT (Green). J) Quantification of the number of 5‐HT+ cells per field in colonic epithelial tissue and quantification of the number of CgA+ cells per field in colonic epithelial tissue. 5‐HT, 5‐hydroxytryptamine; CgA Chromaffin granules protein A; GITT, gastrointestinal transient time. ^**^ *p* < 0.01. n  =  10. Reproduced with permission.^[^ ^192^ ^]^ Copyright 2019, e‐Century Publishing Corporation.

The mechanism of FMT for treating chronic constipation has been explored. Cao et al.^[^ ^135^ ^]^ found that in the intestinal tissues of mice receiving the microbiota of CC patients, the levels of serotonin transporter (SERT) protein were significantly elevated (**Figure** **5**A–F), while serotonin (5‐HT) levels were significantly reduced (Figure 5G–J). These mice all exhibited constipation symptoms, manifested as a decrease in bowel movements per unit time. The results suggest that the dysbiosis of gut microbiota promotes the development of chronic constipation by regulating serotonin transporter proteins in the gut, with a particular focus on the role of SERT. This is a transmembrane transporter involved in the reuptake of excess serotonin from specific sites and in the regulation of gastrointestinal motility. With increased expression in the gut, the levels of 5‐HT decrease, thereby inhibiting intestinal motility and promoting the development of CC. Fu et al.^[^ ^134^ ^]^ supported Cao's experimental results, and Fu also found through fecal microbiota sequencing that the amounts of *Lactobacillus* and *Clostridium* in the constipated group of gast‐ ingesta were significantly reduced, leading to a decrease in gut microbiota diversity, a reduction in probiotics, and a proven increase in bowel frequency. In terms of improving stool consistency and constipation symptoms, *Lactobacillus* has been shown to be more effective,^[^ ^195^ ^]^ while its decrease may lead to insufficient lactic acid secretion, slowing intestinal peristalsis, an increase in pathogenic bacteria, and the accumulation of a large amount of waste affecting normal intestinal metabolism, eventually causing constipation.^[^ ^196^ ^]^ Therefore, dysbiosis of gut microbiota can lead to changes in intestinal motility and damage to the intestinal mucosal protective barrier, ultimately resulting in constipation. Recent studies have shown that FMT can significantly improve symptoms of STC by reshaping the gut microbiome and its metabolite composition.^[^ ^136^ ^]^ After FMT treatment, notable changes were observed in both α‐diversity and β‐diversity of the patients' fecal microbiomes. Based on 16S rRNA microbiome analysis, there was a significant reduction in the abundance of Prevotella/Bacteroides (Bacteroidetes) and Roseburia/Blautia (Firmicutes), while Bifidobacterium (Actinobacteria), Escherichia (Proteobacteria), and Lactobacilli (Firmicutes) increased. These microbial changes were directly related to the reestablishment of a balanced gut ecosystem. In terms of metabolomics, FMT induced significant alterations in key molecules related to metabolic activity in the patients' feces. Post‐FMT, levels of N‐acetyl‐L‐glutamic acid, γ‐L‐glutamyl‐L‐glutamic acid, and glycerophosphocholine significantly increased, suggesting that these metabolites may play important roles in regulating gut cell functions and metabolic pathways. Additionally, serum metabolite profiles also showed significant changes after FMT, with increased levels of L‐arginine, L‐threonine, Ser‐Arg dipeptide, indolepropionic acid, Phe‐Tyr dipeptide, and 5‐L‐glutamyl‐L‐alanine, while levels of erucamide significantly decreased. These changes in metabolites indicate that FMT may regulate host immune responses and gut barrier function through metabolic signaling. Further analysis revealed significant correlations between specific gut microbiota and metabolites. For example, Lactobacillus was positively correlated with L‐arginine, suggesting its potential role in modulating amino acid metabolism to improve gastrointestinal function. L‐threonine was positively correlated with *Anaerovibrio* and *Sediminibacterium*, while negatively correlated with *Phascolarctobacterium*. Erucamide showed a negative correlation with *Sediminibacterium* and *Sharpea*, but a positive correlation with *Phascolarctobacterium*. These relationships suggest that specific microbiota may participate in the regulation of host metabolic pathways through the production or degradation of metabolites. Through KEGG pathway enrichment analysis, the study revealed significant upregulation of the protein digestion and absorption pathway following FMT, particularly key nodes related to amino acid metabolism, such as L‐arginine and L‐threonine. Activation of these pathways is not only associated with improved protein absorption but may also facilitate sodium absorption by gut epithelial cells, enhance mucus secretion, and regulate the electrophysiological properties of gastrointestinal smooth muscles, ultimately improving gut motility.

From 2011 to 2021, more than 1000 patients with STC worldwide were treated with FMT.^[^ ^21^ ^]^ Ding et al.^[^ ^197^ ^]^ standardized FMT for 6 STC patients and the authors observed significant improvements in bowel movements assessment, colonic transit time, constipation‐related symptoms, and quality of life for all patients. Tian et al.^[^ ^198^ ^]^ included data from 20 STC patients aged between 3 and 74 years, who underwent FMT continuously for 12 days via a nasoenteric tube, with results showing significant improvements in gastrointestinal quality of life at the 1st, 2nd, 4th, 8th, and 12th weeks of follow‐up (*p*<0.01). Throughout the entire follow‐up period for FMT procedures, there were no severe adverse events. Additionally, Tian et al.^[^ ^137^ ^]^ demonstrated a clinical cure rate of 73.5% and a clinical remission rate of 14.7% for FMT treatment, with no severe adverse reactions observed.

Therefore, despite the small sample size in the aforementioned experiment, FMT has indeed been proven effective in treating constipation. Washing Microbiota Transplantation (WMT) has been shown to effectively improve refractory FC‐associated therapeutic targets.^[^ ^199^ ^]^ Currently, the combination of FMT with soluble dietary fiber has shown promising advancements in the treatment of STC. A preliminary study included 21 patients with STC, who received FMT for three consecutive days (via nasojejunal tube). Following FMT, they were administered soluble dietary fiber for 4 weeks. The results indicated clinical improvement and relief rates of 66.7% and 42.9% in constipated patients, respectively.^[^ ^200^ ^]^ Targeted FMT therapy for constipation could potentially emerge as a novel option for treating constipation.

### 3.4 Short Bowel Syndrome

Short bowel syndrome (SBS) refers to a condition in which extensive resection of the small intestine results in a reduced effective surface area, leading to a nutritional absorption disorder due to the residual functional intestine being unable to meet the patient's nutritional and physiological needs. Common causes include intestinal torsion, internal and external hernia strangulation, and mesenteric vascular occlusion, which can lead to a significant risk of the entire body being undernourished, resulting in organ function decline, metabolic disorders, decreased immune function, and even the risk of death.^[^ ^201^ ^]^

With the advancement in the treatment of multidisciplinary short bowel syndrome,^[^ ^202^, ^203^ ^]^ new surgical and rehabilitative medical programs have been introduced in addition to the existing medication and nutritional support, leading to improved long‐term efficacy. However, significant morbidity and mortality rates still persist, with adverse reactions that should not be underestimated. Intestinal parenteral nutrition (PN) plays a crucial role in treating the most severe cases of SBS, yet the long‐term use of central venous access devices may lead to adverse effects such as liver disease and infections.^[^ ^204^ ^]^ For SBS patients dependent on PN, the long‐term economic and social pressures, as well as physical torment, have a profound impact on their lives. Therefore, further research and development of more effective therapies are necessary to reduce dependency on PN and enhance the quality of life for SBS patients. In recent years, with the increasing success of fecal microbiota transplantation in the treatment of Clostridioides difficile infection, some have proposed that fecal microbiota transplantation may emerge as one of the novel treatment modalities for short bowel syndrome.

Patients with SBS experience diminished intestinal motility which results in bacterial overgrowth. Furthermore, the reduction in intestinal length leads to decreased associated lymphoid tissue. Chronic inadequate intestinal nutrition can suppress the immunity of the small intestinal mucosa, disrupt intestinal mucosal integrity, and consequently lead to imbalance in the gut microbiota. Concurrently, this dysbiosis exacerbates the development and symptoms of SBS. A study using high‐throughput sequencing techniques revealed significant gut microbiota dysbiosis in patients with short bowel syndrome.^[^ ^205^ ^]^ Piper et al.^[^ ^138^ ^]^ also observed a marked deficiency in commensal Firmicutes such as *Clostridia* in SBS pediatric patients compared to healthy children. The decreased levels of beneficial bacteria result in deficiencies of key intestinal metabolizing enzymes, diminished production of short‐chain fatty acids (which provide energy for intestinal mucosal cells and promote cell growth and repair), and inability to inhibit the growth of certain pathogens (such as *E.coli* and *Streptococcus pneumoniae*). Consequently, the maintenance of intestinal microbial balance is compromised, leading to a variety of diseases and symptoms.

**Figure 6:**
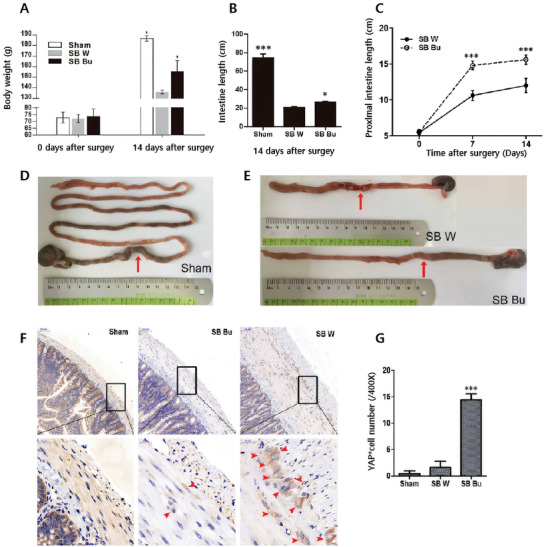
The introduction of oral butyrate supplements can amplify the adaptation of the intestinal smooth muscles in rats undergoing a comprehensive resection of the small bowel. A) Variation in rats’ body weight following surgery throughout the duration of the experiment. B) The overall length of the remaining intestine on day 14 following surgery. (C). Examination and comparison of the proximal portion of the small intestine between the SB W and SB Bu groups on postoperative days 7 and 14. D,E) Visual observation of the residual intestine, displaying alterations in length across various rat groups on the 14th day post‐surgery. The arrows indicate the sites of anastomosis. The FMT‐H succinate activates the expression of YAP. F) The immunohistochemical findings using anti‐YAP in the smooth muscle layers of rats on postoperative day 14 are presented in the figure. The red arrows highlight the presence of a positive signal for YAP. The scale bars in the upper panel represent 100 µm, while those in the lower panel represent 20 µm. G) Bar graph showing quantification of YAP‐positive cells in (A). Values are the means ± SD, ^*^ *p* < 0.05, ^***^ *p* < 0.001, versus SB W. Reproduced with permission.^[^ ^192^ ^]^ Copyright 2019, e‐Century Publishing Corporation.

Butyric acid, as a beneficial short‐chain fatty acid, not only provides energy, renews intestinal epithelium, and suppresses immunity, but also enhances the intestinal adaptation function in SBS patients. The remaining length and function of the small intestine are crucial for improving the quality of life and survival rate of SBS patients. Recent studies have shown that adding beneficial short‐chain fatty acid butyric acid to enteral nutrition in neonatal piglet SBS models significantly improves structural and functional indices of intestinal adaptation.^[^ ^206^ ^]^ Furthermore, Dai^[^ ^192^ ^]^ confirmed that butyrate salts can stimulate the proliferation of human intestinal smooth muscle (ISM) cells in vitro by increasing the yes‐associated protein (YAP) expression pathway (**Figure** **6**F,G), increasing the muscle layer thickness and small intestine length in SBS rats, thereby promoting the adaptability of the residual intestine (Figure 6A–E). Currently, probiotic therapy has shown promising results in treating SBS patients. Pauline^[^ ^139^ ^]^ and others found that oral administration of probiotics such as *lactobacilli* and *bifidobacteria* to piglets (children SBS models) can increase the abundance of Firmicutes and the concentration of short‐chain fatty acids in feces, enrich the diversity of the microbiota, limit potential pro‐inflammatory bacteria in SBS participants, effectively alleviate SBS symptoms, and reduce muscle loss.^[^ ^139^, ^207^, ^208^ ^]^ FMT theoretically can directly alter the recipient's intestinal flora to normalize it, restore a balanced gut microbiome, thus alleviating or treating SBS patients. Davidovics et al.^[^ ^209^ ^]^ successfully treated a case of short bowel syndrome child with D‐lactic acidosis through FMT, where central nervous symptoms improved significantly, and IBS symptoms were successfully relieved. Keep the numbers in brackets unchanged.

Additionally, in light of the distinctive features of SBS gut microbiota (reduced probiotics: such as *Clostridia*, *Bacilli*, and *Bacilliota*), Fourati et al.^[^ ^210^ ^]^ proposed the utilization of a tailored microbial consortium specifically designed for the unique SBS luminal environment, aiming at transplanting specific bacterial groups, namely personalized fecal microbiota transplantation. This offers a new horizon for the advancement of FMT.

**Figure 7:**
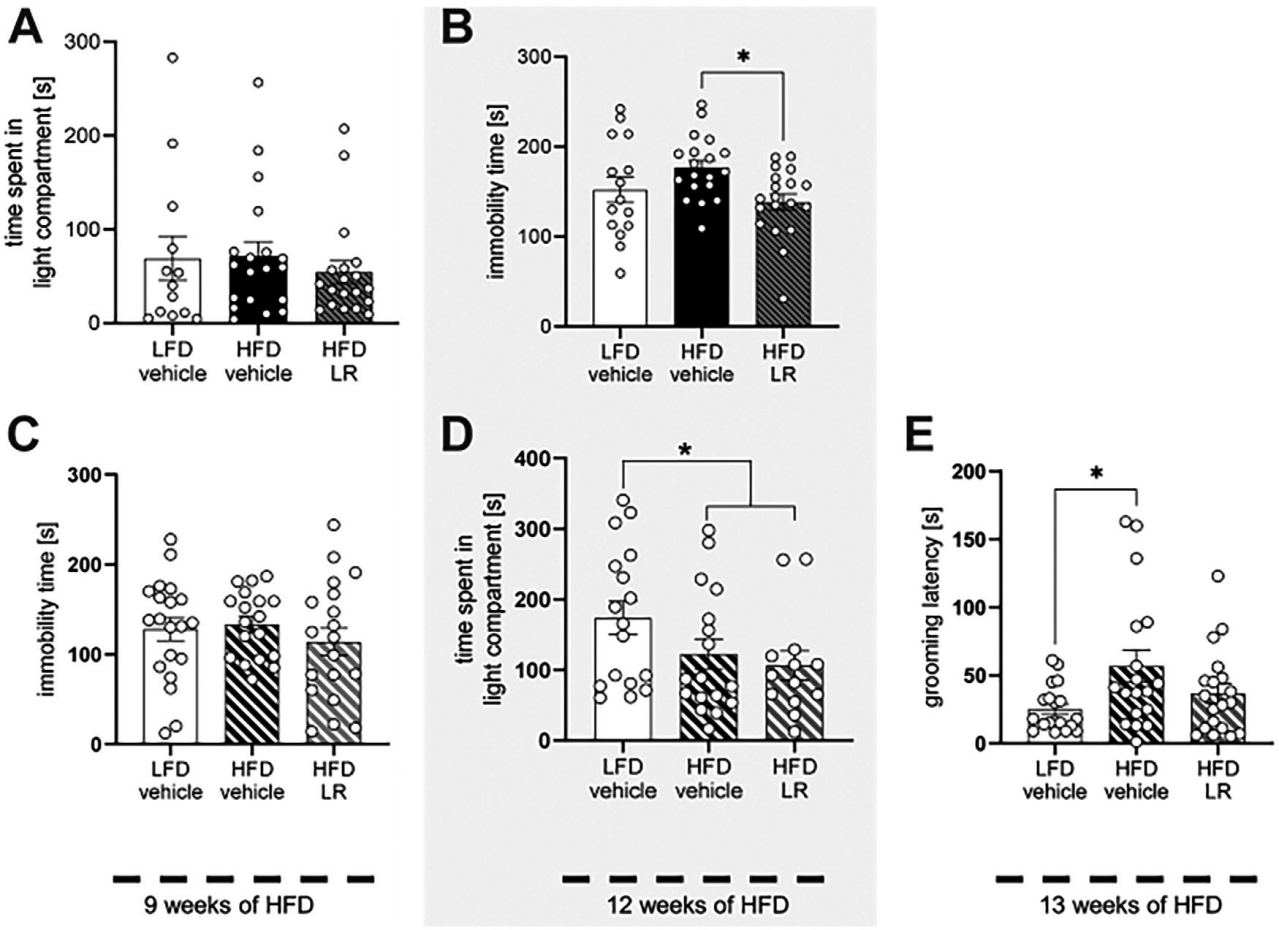
The LR intervention mitigates certain aspects of depressive‐like behavior induced by a high‐fat diet (HFD), yet it does not modulate anxiety. A) Light‐Dark Box Test (LDB) After 9 Weeks of HFD: The time spent by mice in the light compartment was compared between low‐fat diet (LFD vehicle), high‐fat diet (HFD vehicle), and HFD + LR groups. Mice in the HFD group spent significantly less time in the light compartment, indicating increased anxiety‐like behavior. However, LR supplementation did not significantly improve this behavior (no statistical significance). B) Immobility time of male mice after 12 weeks on HFD during the tail suspension test. The results show that the HFD group exhibited significantly increased immobility time compared to the LFD group, indicating a higher level of depressive‐like behavior. There was no significant difference between the HFD + vehicle group and the HFD group, suggesting the vehicle had no effect. However, the HFD + LR group showed a significantly reduced immobility time compared to the HFD group, indicating that LR treatment potentially alleviates depressive‐like behavior. C) The HFD group had increased immobility time compared to the LFD group, confirming that a high‐fat diet increases depressive‐like behavior. However, there was no significant difference between the HFD and HFD + LR groups, suggesting that LR treatment at 9 weeks did not significantly affect depressive‐like behavior or a longer duration of treatment may be needed. D) Light‐Dark Box Test After 12 Weeks of HFD: After 12 weeks of HFD, mice in the HFD group spent significantly less time in the light compartment compared to the LFD group, indicating increased anxiety‐like behavior (*p* < 0.05). LR supplementation significantly improved the behavior of HFD mice, bringing it closer to the LFD group. E) Grooming Latency Test After 13 Weeks of HFD: Mice in the HFD group had significantly longer grooming latencies, indicating lower self‐care motivation. LR supplementation significantly shortened the grooming latency, demonstrating its ability to alleviate the behavioral suppression induced by HFD (*p* < 0.05). Reproduced with permission.^[^ ^211^ ^]^ Copyright 2023, Elsevier.

### 3.5 Irritable Bowel Syndrome

Irritable Bowel Syndrome (IBS) manifests as a chronic biopsychosocial ailment, distinguished by periodic abdominal discomfort and fluctuations in gastrointestinal activity.^[^ ^212^ ^]^ It is estimated that 60–85% of patients with gastrointestinal diseases in clinical settings have mental disorders, and there appears to be a bidirectional relationship between IBS and mental illness. Allegedly, the incidence of depression among IBS patients reaches as high as 84%, with anxiety affecting 44% of individuals. Additionally, a notable 25–30% of those experiencing depression and 10–45% who suffer from anxiety will go on to develop IBS.^[^ ^213^ ^]^ This condition affects 4–10% of the global population, significantly impacting the quality of life.^[^ ^214^, ^215^ ^]^ Currently, the treatment of IBS involves a combination of gastrointestinal motility inhibitors, adsorbents, gut mucosal protectants, and antidepressants, with the therapy process being lengthy and imposing a substantial economic burden on patients, coupled with a high rate of relapse after cessation of medication.^[^ ^216^, ^217^ ^]^ FMT is likely to become an effective and low‐cost treatment for IBS.

The pathogenesis of irritable bowel syndrome involves multiple aspects, including alterations in intestinal motility, visceral hypersensitivity, regulation of intestinal permeability, stress, dysbiosis of gut microbiota, and changes in the microbiota‐gut‐brain (MGB) axis.^[^ ^218^, ^219^ ^]^ The preservation of harmonious equilibrium within the intestinal microbiota culminates in an enduring gastrointestinal ecosystem, intimately engaged in the maturation and proliferation of intestinal mucosal epithelial cells while fostering the advancement of the mucosal immune system.^[^ ^220^ ^]^ Furthermore, the gut microbiota is commonly known as the second brain in humans and plays a pivotal role in overseeing the central nervous system.^[^ ^221^, ^222^ ^]^ Hence, the perturbation of the equilibrium within the gastrointestinal microbiota, coupled with the disruption of the intricate interplay and communication channels between the gut microbiota and the neural, endocrine, and immune systems of the brain, are regarded as fundamental elements contributing to the enduring manifestation of symptoms associated with irritable bowel syndrome.^[^ ^223^, ^224^ ^]^ Numerous studies have demonstrated disparities in the gut microbiota composition between individuals afflicted with IBS and those in sound health,^[^ ^225^, ^226^, ^227^ ^]^ and a thorough and methodical review has revealed that the proportional presence of bacteria in patients with IBS is disrupted when compared to individuals in good health. This manifests as a reduction in beneficial bacteria such as Bifidobacterium and Lactobacillus, and an escalation in pathogenic bacteria like Escherichia coli and Clostridium.^[^ ^140^ ^]^ According to reports by Yang Z, patients with diarrhea‐predominant irritable bowel syndrome (IBS‐D) exhibit lower microbial diversity compared to healthy donors. It is speculated that the dysregulation of the microbiota disrupts the body's balance, impairs the mucosal immune barrier, and consequently leads to the occurrence of IBS.^[^ ^221^, ^222^, ^228^ ^]^

In light of the pivotal role that the gut microbiota plays in facilitating communication between the gut and the brain, scholars have put forth the notion of the microbiota‐gut‐brain axis, commonly referred to as the MGBA.^[^ ^141^, ^142^ ^]^ Countless clinical studies have revealed reciprocal relationships within the MGBA,^[^ ^229^ ^]^ a healthy gut microbiota adeptly acclimates to the host and performs vital biochemical and metabolic processes essential for sustaining normal host functions. Concurrently, signals from the gut microbiota intricately govern the body via pathways of nerve, endocrine, and immune signaling between the gut and the brain, aiming to uphold homeostasis.^[^ ^229^, ^230^, ^231^ ^]^ The study of the brain‐gut axis has emerged as a fertile field of research worldwide, particularly in relation to IBS and the intricate world of the microbiome.^[^ ^232^ ^]^ J F Cryan and colleagues^[^ ^141^ ^]^ Based on a comprehensive analysis regarding the influence of the gastrointestinal microbiome on the cerebral and behavioral aspects, it is proposed that the gut microbiota exerts a regulatory function in relation to anxiety, emotional states, cognition, and pain. The intricate mechanisms underlying this gut‐brain communication encompass the composition of microbial entities, immune stimulation, signaling via the vagus nerve, alterations in tryptophan metabolism, the generation of neuroactive metabolites by specific microorganisms, and the presence of bacterial cell wall sugars.

Lactobacillus rhamnosus (LR), a strain of lactic acid bacteria, has shown significant effects on the central nervous system (CNS) in recent studies, particularly in the production and regulation of γ‐aminobutyric acid (GABA). GABA is the primary inhibitory neurotransmitter in the CNS, playing a crucial role in regulating neuronal excitability, reducing anxiety, promoting relaxation, and improving sleep. Research has demonstrated that LR can influence GABA levels through several mechanisms. First, LR ferments in the gut to produce lactic acid and other short‐chain fatty acids (SCFAs), which can affect brain function through the bloodstream. Specifically, the metabolic activity of LR can activate GABA production in certain brain regions, thereby increasing the overall levels of GABA. This effect is observed in regions such as the cortex, hippocampus, and striatum, which are closely associated with mood, memory, and cognitive functions.^[^ ^233^ ^]^ One study found that long‐term administration of LR (JB‐1) to mice led to region‐dependent changes in GABA B1b mRNA expression in the brain. This suggests that LR not only increases GABA production but may also regulate the expression of GABA receptors, further influencing neurotransmission and behavioral responses.^[^ ^143^ ^]^ Additionally, another study showed that probiotics, including Lactobacillus and Bifidobacterium, could promote the increase of GABA in both the gut and the brain, highlighting the importance of the gut‐brain axis in this process.^[^ ^234^ ^]^ In a study by Schell et al.,^[^ ^211^ ^]^ it was shown that HFD‐fed mice treated with LR had a 22% reduction in immobility time in the tail suspension test (TST), a typical indicator of depressive‐like behavior, suggesting that LR alleviates depression symptoms (Figure 7B). However, no significant effect of LR on anxiety behavior was observed in this study (Figure 7A,B). Given some experimental design limitations, a second cohort was analyzed with an opposite time sequence for anxiety and depression‐like behavior, showing no differences in immobility time between all groups (Figure 7C). HFD feeding reduced the time spent by mice in the light compartment of the light‐dark box test (LDB), indicating anxiety‐like behavior. However, there were no differences in the time spent in the light compartment between the HFD and HFD+LR groups (Figure 7D). To further confirm the impact of LR on depressive behavior, the splash test was conducted. The results showed that LR‐treated mice had a grooming latency similar to that of LFD mice, suggesting that LR treatment may improve self‐care motivation and alleviate depressive behavior induced by a high‐fat diet (Figure 7E). The changes in central GABA receptor expression may be associated with the pathogenesis of anxiety and depression, as well as functional gastrointestinal disorders. It is speculated that LR reduces stress‐induced corticosterone and modulates behavior related to anxiety and depression by regulating GABA receptors. LR might also restore tyrosine hydroxylase, normalizing gene expression in dopaminergic brain regions and modulating signaling pathways associated with mood disorders, thereby alleviating depressive‐like behaviors. Liu et al.^[^ ^235^ ^]^ demonstrated that vagotomy in male mice followed by 14‐day treatment with Lactobacillus rhamnosus significantly reduced anxiety‐like behaviors, further indicating the importance of vagal nerve integrity for LR's anxiolytic and hypothalamic‐pituitary‐adrenal (HPA) axis‐modulating effects. Furthermore, Ford et al.^[^ ^236^ ^]^ found that antidepressants effectively alleviate symptoms of irritable bowel syndrome (IBS), suggesting that Lactobacillus strains may regulate both emotional behavior and central GABA receptor expression through the vagus nerve, potentially providing an effective therapeutic approach for mood disorders.

In recent years, the efficacy and economic benefits of FMT for the treatment of IBS have gained increasing attention from researchers. Over the past decade, nearly 1000 IBS patients have undergone FMT treatment.^[^ ^21^ ^]^ The effects of FMT on IBS symptoms have been inconsistent, with some studies showing improvement in both symptoms and microbiome profiles. For instance, a study by Mizuno et al. (2017) involving 10 IBS patients found that 6 patients showed symptom improvement within 4 weeks of FMT, and this improvement was associated with high levels of *Bifidobacterium* in the donor stool. This suggests that donor stool rich in *Bifidobacterium* may be a key factor for the success of FMT.^[^ ^237^ ^]^ Johnsen et al. (2018) reported that 65% of IBS patients who underwent colonoscopic FMT showed significant symptom relief after 3 months,^[^ ^238^ ^]^ with the recipients' gut microbiome shifting toward that of the donor, including an increase in both alpha(Alpha Diversity: A measure of species richness and diversity within a single sample or specific environment, reflecting the internal complexity of a microbial community.) and beta diversity(Beta Diversity:A measure of the differences in species composition between different samples or ecosystems, used to compare the similarity or dissimilarity of microbial communities across environments.).^[^ ^239^ ^]^ Mazzawi et al. (2018) found that diarrhea‐predominant IBS patients improved in both symptoms and quality of life after receiving fresh stool FMT, with a significant increase in short‐chain fatty acids (SCFAs) and a microbiome profile more closely resembling that of the donor.^[^ ^240^, ^241^ ^]^ Lahtinen et al. (2020) found that whether patients received healthy donor stool (allogeneic transplant) or their own stool (autologous transplant), IBS symptoms were temporarily relieved, with a significant reduction in depression scores in the allogeneic group.^[^ ^242^ ^]^ Additionally, El‐Salhy et al. (2020) used feces from a single healthy donor for FMT, and found that both 30g and 60g doses improved patients' fatigue and quality of life,^[^ ^243^ ^]^ with changes observed in the bacterial microbiome and SCFA levels,^[^ ^244^ ^]^ and this effect was not influenced by gender.^[^ ^245^ ^]^ Holvoet et al. (2021) studied refractory IBS patients and found that 56% of patients reported symptom and quality of life improvements one year after FMT, with recipients' gut microbiomes having higher diversity before FMT, which could potentially serve as a predictor for the success of FMT.^[^ ^246^ ^]^ However, some studies have failed to demonstrate significant efficacy of FMT for IBS symptoms. Halkjær et al. (2018) found that although moderate to severe IBS patients experienced some symptom improvement after 12 days of FMT capsule treatment, the placebo group reported better symptom relief at 6 months.^[^ ^247^ ^]^ In the study by Aroniadis et al. (2019), diarrhea‐predominant IBS patients who received more than 25 FMT capsules did not show significant symptom improvement compared to the placebo group after 3 months.^[^ ^248^ ^]^ These results suggest that individual differences and optimization of treatment strategies should be taken into account when applying FMT for IBS.

The FMT presents a harmonious blend of safety and efficacy, enhancing bacterial diversity, modulating bacterial community distribution, thereby impacting clinical and psychological symptoms; in conclusion, a small subset of IBS patients may benefit from FMT. FMT can be deemed as a promising and prospective therapy for IBS‐D concomitant with anxiety and depression to restore gut microbiota. Future research should elucidate which IBS patients should opt for FMT and which donor microbiota are effective. Additionally, it remains undetermined whether antibiotic pretreatment is necessary, and the frequency of FMT repetition.

### 3.6 Colorectal Tumor

Colorectal cancer (CRC) is a prevalent malignancy in the digestive tract, often necessitating traditional interventions like surgical excision, radiotherapy, and chemotherapy. Mounting evidence indicates a potential association between gut microbiota dysbiosis and the onset of gastrointestinal malignancies.^[^ ^250^, ^251^, ^252^ ^]^ Consequently, researchers are currently investigating the potential uses and mechanisms of Fecal Microbiota Transplantation (FMT) in the management of colorectal tumors. Previous studies have predominantly outlined the pathways through which the gut microbiota influences the progression of cancer, encompassing gut microbial dysbiosis, immune and inflammatory responses, genetic damage, and gut microbial metabolites.

Gut microbiota dysbiosis plays a crucial role in the onset of CRC. Current research indicates that the gut microbiome, tumor microbiome, and immune system interact in complex ways during cancer development. In a healthy state, the gut microbiome is referred to as “eubiosis,” where bacterial diversity is balanced, pro‐inflammatory and anti‐inflammatory cytokines maintain dynamic equilibrium, immune cells and IgA secretion are at appropriate levels, and the mucosal barrier and mucus layer functions are intact.^[^ ^253^ ^]^ However, dysbiosis disrupts these critical parameters, leading to intestinal dysfunction. Dysbiosis not only affects gut health but also disrupts host immune responses and intestinal barrier integrity through various mechanisms.^[^ ^254^ ^]^ It is now believed that gut microbiota dysbiosis may play a dual role in cancer development: on the one hand, it acts as a driver of cancer progression, and on the other, it represents a consequence of tumor progression, reflecting changes in the host immune system and gut microbiome environment.^[^ ^255^ ^]^ Dysregulated microbial communities may promote chronic inflammation in the gut, alter the immune environment, and damage intestinal barrier function, leading to cellular damage, mutations, and carcinogenesis. Additionally, the tumor microbiome's negative impact may exacerbate this imbalance, impairing the host immune system's response to tumors, allowing tumor cells to escape immune surveillance and further promoting cancer progression.^[^ ^254^, ^256^ ^]^ On the other hand, as cancer progresses, the tumor microbiome negatively impacts the gut microbiome, altering its structure and function. This change not only weakens the gut immune response but may also contribute to resistance to chemotherapy and immunotherapy, thereby affecting treatment efficacy.^[^ ^257^, ^258^ ^]^ Notable differences in the gut microbiota between CRC patients and healthy individuals have been identified, including a reduction in commensal bacteria and an increase in carcinogenic bacteria.^[^ ^259^, ^260^ ^]^ The microbial composition of feces and mucosa from CRC patients also shows significant differences.^[^ ^261^ ^]^ Touchefeu et al.^[^ ^262^ ^]^ found that compared to the control group, bacteria such as *F. prausnitzii*, *Barnesiella intestinihominis*, *Alistipes finegoldii*, *Bacteroides eggerthii*, and *Eubacterium siraeum* were significantly reduced in CRC patients. Common bacteria associated with CRC include *Fusobacterium nucleatum*, *Escherichia coli*, *Bacteroides fragilis*, *Enterococcus faecalis*, *Clostridium septicum*, *Enterococcus faecalis*, and *Bacteroides vulgatus.^[^* ^263^ ^]^ Wang et al.^[^ ^264^ ^]^ noted a significant increase in the abundance of *Bacteroides fragilis*, *Enterococcus faecalis*, *Escherichia/Shigella*, *Klebsiella*, *Streptococcus*, and *Peptostreptococcus* in the gut microbiota of CRC patients, while butyrate‐producing *Roseburia* and *Fusicatenibacter* were relatively low. Moreover, certain bacteria such as *Escherichia coli*, *Bacteroides fragilis*, and *Peptostreptococcus anaerobius* have been associated with CRC by activating Th17 cell responses and inducing DNA damage.^[^ ^265^ ^]^

Furthermore, the gut microbiome can participate in tumor formation through immune responses and inflammatory reactions. *Enterotoxigenic Bacteroides fragilis (ETBF)* activates Toll‐like receptors, leading to an increase in inflammatory cytokines such as IL‐6 and TNF‐α, as well as further activation of STAT3 and NF‐kB. ETBF also inhibits the packaging of miR‐149‐3p in exosomes, thereby promoting selective RNA splicing of KAT2A mediated by PHF5A in CRC cells, ultimately promoting CRC cell proliferation.^[^ ^146^ ^]^ Additionally, studies have found a downregulation of the farnesoid X receptor (FXR) in CRC patients, resulting in dysregulated bile acid metabolism and further promoting the colonization of *ETBF*, thereby promoting the occurrence of colorectal tumors.^[^ ^266^ ^]^ It is known that the subspecies gallolyticus of *Streptococcus gallolyticus (Sgg)* is closely associated with colorectal cancer. Recent research by Taylor et al.^[^ ^267^ ^]^ has found that the pathogenicity of *Sgg* is related to the *Sgg* pathogenicity‐associated region (SPAR), which is a chromosome locus. Deletion of this locus significantly reduces the adhesion of *Sgg* to CRC cells and eliminates its ability to stimulate CRC cell proliferation. *Fusobacterium nucleatum* commonly inhabits extensively dysplastic colorectal tissues and adenomas, instigating the secretion of pro‐inflammatory substances such as IL‐6, IL‐8, IL‐1β, TGF‐β, and TNF‐α, thereby fostering the progression of CRC.^[^ ^268^ ^]^ Garrett WS et al.^[^ ^269^ ^]^ have demonstrated that *Fusobacterium nucleatum* adheres to calcium‐binding proteins on the surface of CRC cells via adhesin FadA and Fap2. This inhibits the activity of tumor‐infiltrating lymphocytes and natural killer cells, while activating the oncogenic Wnt/β‐catenin signaling pathway, ultimately contributing to the promotion of CRC. Moreover, a newly published study by Flukiger^[^ ^270^ ^]^ indicates that certain intestinal bacteriophages can enhance the effectiveness of anti‐tumor immunotherapy by triggering the development of symbiotic‐specific memory T cells that can cross‐react with tumor antigens.

Furthermore, the gut microbiota has the ability to generate proteins, molecules, and secondary metabolites that result in direct DNA damage, thereby interacting with the host DNA and eliciting mutations.^[^ ^271^ ^]^ *ETBF* can cause chronic inflammation and produce extracellular superoxides and hydrogen peroxide, leading to DNA damage and chromosomal instability.^[^ ^147^ ^]^ In a mouse model of CRC, it is known that enterotoxigenic B. fragilis and pks+ Escherichia coli have a synergistic carcinogenic effect. The co‐colonization of *ETBF* and *pks+ E. coli* increases tumor burden. *ETBF* promotes mucin degradation, which favors the colonization of *pks+ E. coli*, resulting in genetic toxicity. Perhaps in the future, the detection of *ETBF* and *pks+ E. coli* can be used to predict colorectal cancer.^[^ ^272^ ^]^

In the course of CRC initiation and progression, the metabolic products of the gut microbiota also play a significant role. Within the human body, the gut microbiota engages in intricate and dynamic metabolic activities, providing not only the energy and nutrients necessary for its own growth and reproduction, but also generating numerous metabolites that enter the human system. Compounds such as ammonia, phenols, and hydrogen sulfide produced by the gut microbiota have been implicated in the development of CRC through the induction of chronic inflammation and DNA damage.^[^ ^75^, ^148^, ^149^ ^]^ Furthermore, nitrogen‐containing products generated by gut microbial metabolism, such as N‐nitroso compounds (NOCs), promote carcinogenesis through DNA alkylation, exerting carcinogenic effects.^[^ ^273^ ^]^ T These studies emphasize the pivotal significance of dysbiosis, inflammation, or immune suppression in the genesis of colorectal cancer by the gut microbiota, along with the direct engagement of proteins, molecules, and secondary metabolites that possess the capacity to directly instigate DNA impairment. Furthermore, the metabolic byproducts of the gut microbiota can play a direct role in the initiation of cancer.

FMT is currently a hot topic in cancer treatment. Researchers from various countries are actively exploring its clinical efficacy. In a study by Yu et al.,^[^ ^150^ ^]^ FMT was experimentally applied based on a mouse model of CRC with dysregulated gut microbiota. The researchers transferred gut microbiota from healthy mice to CRC mice through fecal enema. The study results showed that FMT significantly reversed the severe dysbiosis of gut microbiota in CRC mice. It also increased the proliferation of immune cells that directly kill cancer cells, including CD8+ T and CD49bNK cells. FMT reduced the generation of immunosuppressive cells, specifically Foxp3+Tregs cells. It also regulated the expression of inflammatory cytokines in CRC mice, leading to downregulation of IL1a, IL6, IL12a, IL12b, IL17a, and upregulation of IL10. In this way, FMT effectively inhibited the development of CRC.

**Figure 8:**
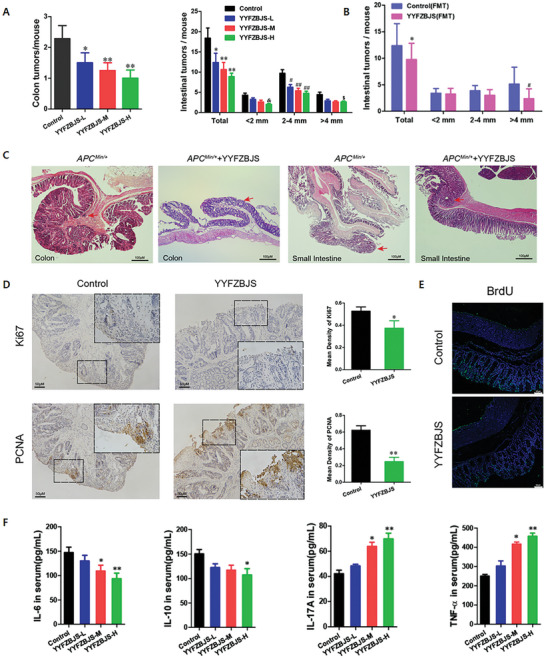
Inhibitory Effects of YYFZBJS on Colorectal Tumors and Inflammatory Factors in Mice. A) Effect of YYFZBJS on Tumor Numbers in the Colon and Small Intestine: Left panel shows that YYFZBJS at low (YYFZBJS‐L), medium (YYFZBJS‐M), and high (YYFZBJS‐H) doses significantly reduced the number of colon tumors in mice (^*^ *p* < 0.05, *p* < 0.01). Right panel illustrates the effect of YYFZBJS on tumors of different sizes in the small intestine: YYFZBJS treatment, especially at the high dose, significantly reduced the total number of tumors as well as the number of tumors in the 2–4 mm and >4 mm size ranges (*p* < 0.01, ##*p* < 0.01). B) Effect of Fecal Microbiota Transplantation (FMT) on Small Intestinal Tumor Numbers: The FMT group showed a significant reduction in the total tumor number and the number of 2–4 mm tumors, with the combination of YYFZBJS and FMT demonstrating a more pronounced therapeutic effect (^*^ *p* < 0.05, *p* < 0.01, #*p* < 0.05). C) Left: A depiction of a standard adenomatous polyp observed in ApcMin/+ mice, exhibiting an advanced state of dysplasia and the presence of carcinoma in situ. Middle: An adenomatous intestinal polyp, portraying the initial invasion of neoplastic glands into the muscular layers, which is frequently observed in ApcMin/+ mice. Right: A diminutive polyp featuring residual dysplastic glands in close proximity to the surface epithelium. This characteristic regressive morphology of intestinal cancer can be identified throughout the entirety of the mice's intestine. The red arrows denote the presence of adenocarcinoma cells. The magnification bars indicate a scale of 100µm. (D&E). Immunohistochemical staining utilizing an antibody targeting PCNA, Ki67, and BrdU was conducted in both the control group and the group undergoing YYFZBJS treatment. The magnification bars were set at 50µM. The data presented are expressed as means ± standard deviation from a total of 8 animals per experimental group, with Welch's correction applied and a one‐tailed *t*‐test performed. D) Changes in Cell Proliferation Markers (Ki67 and PCNA) Expression: YYFZBJS significantly decreased the expression of Ki67 and PCNA in colorectal tumor tissues, indicating its effective inhibition of cell proliferation activity (^*^ *p* < 0.05, *p* < 0.01). (E) BrdU Labeling Analysis: BrdU staining results showed a significant reduction in BrdU‐positive cells in the colon tissues of YYFZBJS‐treated mice, further supporting its inhibitory effect on cell proliferation. (F).IL‐6, IL‐10, IL‐17A and TNF‐α levels in PBMC of ApcMin/+ were evaluated using ELISA. Inflammatory Factor Level Analysis: YYFZBJS significantly reduced the levels of pro‐inflammatory cytokines IL‐6 and TNF‐α in the serum (^*^ *p* < 0.05, *p* < 0.01), while increasing the level of the anti‐inflammatory cytokine IL‐10 (*p* < 0.05). However, high‐dose YYFZBJS significantly elevated the level of IL‐17A (*p* < 0.01), suggesting a complex regulatory role on Th17 cell‐related pathways. The intragastric administration of YYFZBJS‐L/M/H were taken at the doses of 3.825, 7.65, and 15.3 g kg^−1^ according to HED (human equivalent dose). One group was gavaged fecal samples from healthy controls (Control‐FMT), while the other group was gavaged fecal samples from people who eating YYFZBJS (YYFZBJS‐FMT). The data are presented as the mean ± SD from at least three experiments. ^*^ *p* < 0.05, ^**^ *p* < 0.01 versus control. Reproduced with permission.^[^ ^249^ ^]^ Copyright 2020, BioMed Central.

FMT has also made significant breakthroughs in traditional Chinese medicine. Hua Sui et al.^[^ ^249^ ^]^ fed feces samples from mice treated with a traditional Chinese medicine called Yi Fu Zi Zi Bai Jiang San (YYFZBJS) to C57BL/6 J ApcMin/+ mice, an animal model for intestinal tumor formation. The study found that YYFZBJS reduced the number of adenomas in mice (**Figure** **8**A) and even completely eradicated early‐stage colon cancer histologically (Figure 8C). The total number of intestinal tumors in mice also significantly decreased (Figure 8B). Additionally, Ki67 and PCNA nuclear expression levels in the colonic polyp epithelium were reduced after YYFZBJS treatment (Figure 8D,E), indicating inhibited tumor cell proliferation. Moreover, the expression of IL‐6 and IL‐10 in mouse peripheral blood mononuclear cells (PBMCs) decreased significantly, while the expression of IL‐17A and TNF‐α increased (Figure 8F). These findings suggest that this process also modulated the expression of related inflammatory factors. In summary, YYFZBJS treatment prevented the occurrence and progression of Apc tumors and enhanced immune function.

Furthermore, some researchers have found that FMT has positive effects in mitigating the side effects of immunotherapy in cancer patients. For example, radiation therapy often damages the intestinal epithelial cells. Cui et al.^[^ ^274^ ^]^ found that transplanting fecal microbiota into the small intestines of irradiated mice improved epithelial integrity and promoted angiogenesis without promoting tumor growth. This increased the survival rate of the mice. Therefore, FMT is considered a potential radioprotective agent that can improve the prognosis of cancer radiation therapy.

Recent studies have shown that FMT may alleviate diarrhea and intestinal mucositis induced by the FOLFOX chemotherapy regimen (comprising 5‐fluorouracil, leucovorin, and oxaliplatin) in a CRC mouse model by modulating the gut microbiome. In this experiment, BALB/c mice were implanted with CT26 colorectal adenocarcinoma cells and subsequently treated with FMT for 7 days, including 5 days of FOLFOX chemotherapy followed by 2 days post‐treatment. The results revealed that FOLFOX treatment significantly induced diarrhea and intestinal damage in the mice, while FMT effectively alleviated these symptoms and reduced the severity of intestinal mucositis. After FOLFOX treatment, a series of cellular and molecular changes were observed, including a decrease in goblet cells and tight junction protein zonula occludens‐1, and an increase in apoptotic and NF‐κB‐positive cells. Furthermore, FOLFOX treatment led to upregulation of Toll‐like receptors (TLRs), MyD88, and IL‐6 levels, which were significantly attenuated following FMT treatment, indicating that FMT exerted a protective effect by modulating immune responses and inflammatory signaling pathways. Specifically, FMT alleviated intestinal inflammation by suppressing the overactivation of the TLR‐MyD88‐NF‐κB signaling pathway, thereby mitigating FOLFOX‐induced intestinal damage. In addition, FMT restored the dysregulated gut microbiome composition following FOLFOX treatment, further enhancing intestinal barrier function and stabilizing the immune system. Notably, FMT did not induce bacteremia, suggesting its high safety profile. This study demonstrates that FMT not only reduces the toxicity of chemotherapy drugs to the gut but may also enhance CRC patients' tolerance to FOLFOX therapy by restoring gut microbiome balance and reducing chemotherapy‐related side effects. Encouragingly, FMT may also exert synergistic effects with other therapies, collectively inhibiting CRC cell progression.^[^ ^275^ ^]^ Zhao et al.^[^ ^276^ ^]^ conducted a single‐arm, open‐label, phase II clinical trial in which the combination of fruquintinib (a small‐molecule VEGF receptor tyrosine kinase inhibitor) and tislelizumab (a PD‐1 monoclonal antibody inhibitor) with fecal microbiota transplantation was employed to treat patients with proficient mismatch repair (pMMR) and microsatellite‐stable (MSS) stage IV colorectal cancer. The study confirmed the safety and controllability of this treatment regimen, as well as its ability to improve survival in microsatellite‐stable metastatic CRC patients. Specifically, the treatment extended the median progression‐free survival by 9.6 months, increased the median overall survival by 13.7 months, and improved the overall response rate and disease control rate by 20% and 95%, respectively. Additionally, fecal microbiota transplantation significantly enhanced the efficacy of tumor immunotherapy, leading to marked improvements in clinical outcomes.

Multiple studies have revealed the potential of FMT in alleviating various aspects of cancer associated with intestinal dysbiosis and cancer treatment‐related complications. However, the quality of evidence for FMT in cancer treatment remains generally low, necessitating high‐quality clinical data and large sample randomized controlled trials to further investigate whether FMT can serve as a safe intervention for cancer treatment. Further in‐depth research into the mechanisms between gut microbiota and colorectal cancer is crucial. Additionally, more research and validation are still needed to clarify the safety, efficacy, and optimal application of FMT in colorectal tumor therapy. This will aid in the development and translation of potential cancer prevention strategies based on gut microbiota.

**Figure 9:**
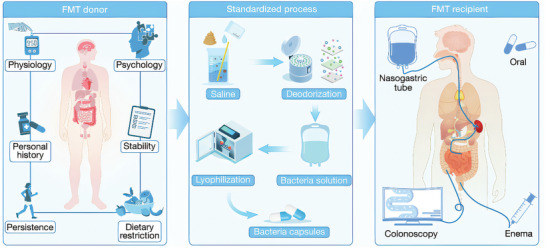
Schematic Representation of the Fecal Microbiota Transplantation (FMT) Process. A): FMT Donor Selection. Depicts the evaluation process for physiological and psychological health, including personal medical history and dietary restrictions. B): FMT Processing Steps. Details the standardized procedures from sample collection from the donor, saline processing, deodorization, to lyophilization, and encapsulation into capsules. C): FMT Recipient Administration Methods. Illustrates various administration routes including oral capsules, nasogastric tube, direct colonoscopic infusion, and enema. Reproduced with permission.^[^ ^277^ ^]^ Copyright 2024, hLife.

## 4 Factors Affecting FMT Efficacy and Key Elements of Success

The process of FMT, as illustrated in the diagram (Figure 9), includes donor screening, fecal material production, and transplantation. Donor selection is a critical step to ensure both the safety of the donor and the quality of the fecal material. The donor screening process encompasses medical history review, lifestyle assessment, infectious disease testing, and gut microbiome analysis. While screening standards are primarily based on guidelines from Europe and the United States, it is important to note the differences in body mass index, dietary habits, and gut microbiome composition between Asian and Western populations. As a result, China has developed a more comprehensive donor screening system that covers six key dimensions: physiological health, psychological status, personal medical history, microbiome stability, long‐term donor sustainability, and dietary tolerance.^[^ ^278^ ^]^ After the donor passes rigorous screening, the process of fecal material production for FMT becomes crucial. This includes the collection of feces under specific conditions, processing in an anaerobic environment, and the preparation of the microbiota solution, which is then stored at ‐80°C to maintain its viability. The production process must be standardized and automated to ensure microbial abundance and activity, improving the efficacy of FMT while reducing potential risks. Proper management of donors is also essential, including obtaining informed consent, conducting regular health assessments, and maintaining records of donation frequency. Additionally, establishing a traceability system is vital for tracking the safety and efficacy of FMT, ensuring that all materials and information are traceable.

Through these meticulous steps, high‐quality fecal material can be provided for effective FMT treatment. Once processed, the fecal material is ready for transplantation. The final step in FMT is the introduction of the processed fecal material into the patient's body. The delivery route can be tailored to the patient's specific condition, with fecal material being administered either through the upper or lower gastrointestinal tract. Upper gastrointestinal delivery involves the use of gastroscopy, nasogastric tubes, nasojejunal tubes, gastrostomy tubes, or oral capsules. Lower gastrointestinal delivery typically involves colonoscopy, sigmoidoscopy, or retention enema for fecal administration. According to current research, colonoscopy should be the preferred route for FMT administration.^[^ ^279^ ^]^ Available capsule formulations include Microbiome Ecosystem Therapeutic 2 (MET‐2),^[^ ^280^ ^]^ Rebyota (RBX2660), and Vowst (SER‐109).

Although FMT has demonstrated significant efficacy in treating rCDI, its application in chronic non‐communicable diseases continues to face numerous challenges, such as inconsistent research data and the complexity of the treatment process. To advance the use of FMT in the treatment of these diseases, there is an urgent need to delve into the key factors influencing the colonization of microbial communities in the recipient's gut, as well as analyze the impact of donor selection, recipient characteristics, and treatment protocols on clinical outcomes. Additionally, innovation in diagnostic techniques and therapeutic approaches will also enhance the further development of microbiota‐based therapeutic strategies.

### 4.1 Factors Influencing Effects

#### 4.1.1 Donor‐Related Factors

The selection of the donor significantly impacts the effectiveness of FMT, primarily in terms of the donor's health status, microbial diversity, and the abundance of specific microbiota. Studies have shown that healthy donors typically possess higher microbial diversity, which contributes to improved FMT efficacy, particularly in the treatment of rCDI and other diseases.^[^ ^108^ ^]^ Donor α‐diversity has been found to be closely associated with the success rate of FMT, as higher diversity provides more comprehensive microbial support and enhances the colonization ability of the recipient's gut microbiota.^[^ ^281^ ^]^ However, when the donor's microbiota is unstable, it may introduce the risk of transmitting harmful microorganisms, thereby affecting treatment outcomes.^[^ ^282^ ^]^ Therefore, donor screening should not only focus on infectious pathogens but also consider the health status of the microbiota to ensure its quality.^[^ ^283^ ^]^ Furthermore, standardizing microbiota matching and treatment protocols helps improve FMT success rates and reduce variability in treatment outcomes.^[^ ^284^ ^]^

The screening of donors are crucial for the safety and efficacy of FMT. Current clinical guidelines for FMT emphasize the importance of screening donors to ensure that transplant materials are free from infectious pathogens.^[^ ^23^ ^]^ For instance, the FDA mandates the inclusion of MDRO screening in research protocols for FMT to prevent the transmission of SARS‐CoV‐2. Recent cases and studies suggest that FMT may lead to the long‐term transmission or eradication of potentially harmful microbial features,^[^ ^26^, ^27^ ^]^ which could depend on various factors such as the age of the recipient or the frequency of fecal infusions.^[^ ^24^ ^]^ Further research is needed to enhance the safety of FMT, such as assessing the risk of specific microbial feature transfer through metagenomic tracking, and understanding how the dissemination of these features may trigger the development of specific diseases. Additionally, the health status of donors, including conditions like cancer and chronic diseases, is also a factor that needs to be considered in the screening process.^[^ ^23^ ^]^

The ecological parameters and taxonomic composition of the donor microbiota are associated with the clinical success of FMT. The alpha diversity of the donor,^[^ ^285^ ^]^ the abundance of specific taxa,^[^ ^180^, ^286^ ^]^ as well as the composition of the gut virome^[^ ^287^ ^]^ and mycobiome^[^ ^288^ ^]^ may all influence the efficacy of FMT. Individual taxa are not a consistent predictor of clinical success, given the complexities of the microbial community in the natural course of the disease.^[^ ^289^ ^]^ Future research will need to further explore matching models based on hierarchical analytical processes or machine learning methods^[^ ^290^ ^]^ to identify the most suitable donors for specific recipients, thereby enhancing the clinical efficiency of FMT.

#### 4.1.2 Receptor‐Related Factors

The gut microbiota is a complex niche shaped by host and environmental factors in synergy. These driving forces converge to facilitate the resilience of gut microbial communities following acute disturbances, such as FMT. Post‐FMT, these host factors will reconfigure the transferred microbial community, achieving a new host‐microbe equilibrium.

The intestinal ecosystem of the recipient is influenced by host genetic and immune factors, which reshape the microbiota after FMT. Genetic factors serve as a potent driving force for the composition of the gut microbiota,^[^ ^291^ ^]^ while the immune response plays a crucial role in the interaction between the host and the microbiota.^[^ ^292^ ^]^ The timing of FMT is paramount for the successful colonization of donor strains,^[^ ^293^ ^]^ as evident in significant and enduring implantation in the intestines of mice during the weaning and neonatal periods.^[^ ^294^ ^]^ During the process of FMT colonization, particular attention should be paid to the host's mucosal immune status and levels of inflammation.^[^ ^8^ ^]^ Controlling mucosal inflammation before performing FMT may facilitate the establishment of beneficial microbial communities adapted to a non‐inflammatory environment, thereby aiding in mucosal healing.^[^ ^295^ ^]^ These findings underscore the importance of considering the host's genetic background and immune status in FMT treatment plans.

The resilience of the recipient gut microbiota is crucial for the success of FMT, depending on the stability of the microbial community, its competitive ability in nutrient supply, and its capacity to adapt to the new redox state of the mucosal environment.^[^ ^296^ ^]^ The baseline microbial diversity of the recipient is associated with the clinical outcomes of FMT, but this relationship may vary depending on the disease, as the fecal microbial diversity of individuals with metabolic syndrome is lower,^[^ ^22^ ^]^ while successful FMT in IBD recipients is associated with higher baseline bacterial diversity.^[^ ^176^ ^]^

#### 4.1.3 Employment Agreement

The working protocol of FMT encompasses several crucial steps, such as antibiotic pretreatment and intestinal lavage, which are paramount to the success of FMT.

Pre‐treatment with antibiotics is a standard practice in patients with rCDI,^[^ ^23^ ^]^ but the use of antibiotics in non‐communicable diseases may require more caution.^[^ ^297^ ^]^ Antibiotic pre‐treatment may serve as a preparatory therapy, beneficial for the colonization of donor microbiota,^[^ ^22^ ^]^ however, the selection, dosage, and duration of antibiotics can impact the engraftment rate.^[^ ^298^, ^299^ ^]^

Bowel preparation is a necessary step before colonoscopy, but there is still controversy regarding the optimal method and timing. Some studies suggest that bowel preparation can reduce bacterial load and may benefit microbiome implantation.^[^ ^291^ ^]^ However, for certain patients with IBD, bowel preparation may induce mucosal irritation and inflammation. A study by Péter et al.^[^ ^300^ ^]^ showed that bowel preparation might alter the fecal microbiota composition in IBD patients, including a decrease in the abundance of *Bifidobacteriaceae*, *Enterobacteriaceae*, *Veillonellaceae*, and *Pasteurellaceae*, while the abundance of *Streptococcaceae* increased. Drago et al.^[^ ^301^ ^]^ also reported a significant reduction in the number of probiotics and the abundance of *Lactobacillaceae*. The reduction in the α‐diversity of these gut microbiota and changes in their abundance may play a potential role in disease exacerbation and may reduce the efficacy of newly initiated biologic therapies.^[^ ^300^ ^]^ Therefore, it is recommended to accurately define the indications for colonoscopy to avoid disease flare‐ups caused by changes in the microbiome. Additionally, given the need for possible treatment adjustments and the risk of disease relapse, the timing of colonoscopy should be emphasized. Future research should validate these findings, further clarify potential treatment recommendations and their consequences, and establish the optimal bowel preparation strategy for patients.

The frequency of fecal infusion, volume of infused feces, as well as the donor strategy used, are among the various factors that can influence the efficacy of FMT. In patients with CDI, repeated FMT has been shown to increase the cure rate,^[^ ^25^ ^]^ while in chronic non‐communicable diseases, consecutive FMT has also been proven to be beneficial.^[^ ^302^ ^]^ The quantity of fecal infusion is associated with the clinical outcomes of FMT,^[^ ^284^ ^]^ but the assessment methods for fecal weight are decreasing, shifting the focus toward the total amount of viable microorganisms in the feces.^[^ ^303^ ^]^ The multi‐donor FMT strategy involves combining fecal material from different donors to enhance the diversity of the infusion solution,^[^ ^304^, ^305^ ^]^ and has shown better efficacy in some studies, however, the long‐term effects and safety of this approach still require further investigation.

FMT can be administered through various routes, including endoscopy, enema, capsules, and others. Encapsulation of FMT has demonstrated efficacy comparable to colonoscopic administration in preventing rCDI, and may be suitable for long‐term microbiota modulation.^[^ ^302^ ^]^ The role of diet in FMT is increasingly recognized, with the diet of both donors and recipients potentially influencing the outcomes of FMT.^[^ ^243^ ^]^ Specific diets, such as high‐fiber diets^[^ ^306^ ^]^ or anti‐inflammatory diets,^[^ ^304^ ^]^ may synergize with FMT to improve patients' clinical symptoms. These findings offer new perspectives on utilizing diet as an adjuvant to FMT and developing combined approaches for therapeutic microbiota modulation.

### 4.2 Key to Success

In order to elucidate the success of FMT, a comprehensive evaluation of the gut microbiota is crucial. This necessitates the use of high‐throughput sequencing methods such as WGS, particularly shotgun metagenomics(A high‐throughput sequencing technique that randomly sequences all genomic DNA in a sample, allowing for comprehensive analysis of the taxonomic composition, functional genes, and metabolic potential of microbial communities with high resolution.), as it offers a higher taxonomic resolution and insight into the functional potential of the microbial community.^[^ ^307^ ^]^ Furthermore, determining the extent of strain engraftment is key in FMT studies, which can be achieved through the analysis of the microbiota composition of both donors and recipients. Despite the lack of standardized methods for assessing strain engraftment currently, operational definitions based on strain‐specificity and persistence are instrumental in more accurately evaluating microbial engraftment.

In assessing the clinical and microbiological success of FMT, it is important to understand the relationship between clinical success and the implantation of the microbiota.^[^ ^308^ ^]^ While some studies have suggested a correlation between microbiota implantation and clinical success, this finding has not been replicated in all studies.^[^ ^309^ ^]^ The persistence of microbiota implantation and its impact on the duration of clinical success remain a question that requires further investigation.^[^ ^298^ ^]^ To gain a better understanding of the effects of FMT, larger‐scale and more intricately designed studies are needed, along with longitudinal sampling at multiple time points post‐FMT. Additionally, in order to advance FMT as a therapeutic option for non‐communicable diseases, certain cultural barriers need to be overcome, including the lack of microbiota analysis and the perception of FMT as a one‐time therapy.

Donor selection plays a crucial role in Fecal Microbiota Transplantation (FMT), where microbial profiling and machine learning can aid clinicians in choosing the optimal donor for each patient.^[^ ^298^, ^310^ ^]^ Despite the potential demonstrated by FMT in treating non‐communicable diseases, utilizing it as a long‐term therapy for chronic conditions still faces challenges, including the instability of fecal material, safety concerns with prolonged treatment, difficulties in donor recruitment, and unknown drivers of FMT treatment efficacy. To overcome these challenges, further research and the development of novel microbiota‐based therapeutic approaches are necessary.

## 5 Discussion and Conclusion

With the continuous advancement of research on FMT, an increasing amount of clinical data is supporting its potential in the treatment of various gastrointestinal conditions. Ongoing clinical trials indicate that FMT holds significant promise as a therapeutic option. Table 2 provides an overview of current research on the therapeutic potential of FMT in treating multiple intestinal diseases. Nevertheless, for most illnesses, it remains unclear whether alterations in the microbiota directly cause the condition, or if they are merely a consequence of the disease. If changes in the microbiota influence the progression of diseases, then modifying the microbiota could have a positive impact on treatment. However, in most cases, individual microbes are seldomly pathogens or missing beneficial strains. Therefore, FMT introduces the characteristics of an overall healthy gut microbiota, providing unique advantages compared to prebiotics and probiotics. By analyzing the impact of the microbiota on diseases, FMT can serve as a tool to delve deeper into causal relationships, thereby enhancing our comprehension of the pathogenesis and progression of illnesses.

As the liver and intestines not only share anatomical homology but also closely interact in terms of metabolism and immune function. Recent studies have shown that GM can mediate the occurrence and development of various liver system diseases through the gut‐liver axis. The gut‐liver axis is a bidirectional relationship established on the basis of the portal vein between the intestines and the liver, regulating the metabolism and immune response of the intestines and liver through nutrients, metabolites, bile acid metabolism, shaping the structure and function of the microbial community.

As of December 17, 2024, a total of 70 clinical trials related to the treatment of liver diseases with FMT have been registered on ClinicalTrials.gov. These trials focus on conditions such as hepatic encephalopathy (HE), alcoholic hepatitis (AH), cirrhosis, non‐alcoholic fatty liver disease (NAFLD), and non‐alcoholic steatohepatitis (NASH) (**Table** **3**). Research has shown that, in animal models, FMT significantly improves lipid metabolism in NAFLD, primarily through probiotics such as Bifidobacterium, which convert polysaccharides into monosaccharides and produce short‐chain fatty acids (SCFAs) like acetate, propionate, and butyrate. These metabolites help regulate the gut microbiota of liver disease patients, decrease intestinal permeability, and reduce the transfer of endogenous ethanol and endotoxins to the liver, thereby alleviating liver damage.^[^ ^311^, ^312^, ^313^ ^]^ Further data from randomized clinical trials indicate that FMT can particularly improve treatment outcomes in lean NAFLD and NASH patients. This is achieved by modulating microbial dysbiosis, improving gut permeability, and regulating hepatic DNA methylation, which reduces hepatic fat accumulation.^[^ ^313^, ^314^, ^315^ ^]^ Moreover, Philips et al. demonstrated that a 7‐day course of FMT significantly increased one‐year survival rates in patients with severe alcoholic hepatitis (SAH) who were unsuitable for steroid treatment (87.5% versus 33.3%).^[^ ^316^ ^]^ FMT has also been shown to be a safe and effective treatment for cirrhosis, improving microbial dysbiosis, reducing hospitalization rates and cognitive decline, and preventing hepatic encephalopathy events.^[^ ^317^ ^]^ The gut microbiome plays a critical role in the pathogenesis of cirrhosis and its complications.^[^ ^318^, ^319^ ^]^ A study by Wang et al.^[^ ^320^ ^]^ found that FMT improved behavior, hepatic encephalopathy scores, and spatial learning ability in rats, while alleviating liver and intestinal mucosal damage. However, literature also reports a case of a patient who died from Escherichia coli bacteremia following FMT, highlighting the importance of carefully weighing the benefits and risks when considering FMT in liver disease patients, particularly those with severe liver failure and compromised intestinal barriers.^[^ ^321^ ^]^

**Table 3:** FMT Clinical Trials Registered on ClinicalTrials.gov.

Category	Disease category	Number of trials	List of NCT numbers
FMT	Hepatic Encephalopathy (HE)	12	NCT05229289, NCT06368895, NCT06040814, NCT03439982, NCT03420482, NCT02255617, NCT05669651, NCT05241351, NCT03796598, NCT02366547, NCT04155099, NCT04014413
FMT	Alcoholic Hepatitis (AH)	11	NCT02585592, NCT03827772, NCT0307964, NCT02458079, NCT04758806, NCT05548452, NCT05006430, NCT03091010, NCT05448144, NCT04014413 NCT05229289
FMT	Cirrhosis	15	NCT04330469, NCT04842539, NCT0495122, NCT02862249, NCT03416751, NCT03014505, NCT04932577, NCT02019784 NCT03152188, NCT06533852, NCT03796598 NCT04591522 NCT06461208 NCT06478602 NCT06368895
FMT	NAFLD and Related Complications	10	NCT02496390, NCT03648086, NCT05494954, NCT04465032, NCT05607745, NCT04371653, NCT06024681 NCT05821010, NCT05622526, NCT02721264
FMT	NASH and Related Complications	6	NCT02469272, NCT03803540, NCT05821010, NCT05622526, NCT02721264, NCT02868164
FMT	Liver Transplant	4	NCT04621812, NCT02223468, NCT03507140, NCT03666312,
FMT	Liver Failure (Chronic and Acute)	4	NCT03363022, NCT05170971, NCT02689245, NCT04431375,
FMT	Hepatitis B Virus Infection and Related Complications	4	NCT03429439, NCT04431375, NCT03437876 NCT02689245
FMT	Liver Cancer	4	NCT05750030, NCT04303286, NCT05690048 NCT06643533

An interesting finding in research has revealed that the abundance of bacteriophages in FMT is significantly higher (1‐10 times that of bacterial abundance). The transfer of intestinal bacteriophages may be a crucial driving factor for the efficacy of FMT, with the feature being that when the phage composition resembles that of the donor post‐FMT, the cure rate of FMT is higher.^[^ ^322^ ^]^ This highly personalized viral colonization represents a pattern based on specific donor‐recipient pairs.^[^ ^323^ ^]^ Future studies are necessary to better characterize bacteriophages and understand their potential mechanisms in FMT.

Currently, the causal relationship between FMT and disease treatment remains a subject of significant debate. To address this, germ‐free animal systems, such as germ‐free mice, can be established for effective validation. Germ‐free mice are bred in a sterile environment, devoid of any microbiota, providing an uncontaminated platform to study the effects of single or multiple microorganisms on host physiological functions. By transplanting human fecal samples into these germ‐free mice, phenotypic changes, such as body weight, gut barrier function, and immune response, can be observed without interference from other microorganisms, thus allowing for the inference of the microbiome's impact on host health. Since 2006, when Ley et al. first established the causal relationship between the microbiome and obesity using a germ‐free mouse model through FMT,^[^ ^324^ ^]^ germ‐free animal systems have become crucial tools for elucidating the functions of gut microbiota.^[^ ^325^ ^]^ By combining germ‐free animal models, FMT, and multi‐omics technologies, researchers can analyze microbiome changes in major human complex diseases, identify key functional microbes, explore their mechanisms of action, and achieve clinical translation. This approach also facilitates the development of new therapeutic strategies, such as the selection, cultivation, identification, and utilization of FMT formulations or individual strains, with the potential to evaluate these microorganisms in disease treatment.

In summary, FMT stands as a promising therapeutic strategy for various microbiome‐related disorders. Nevertheless, apart from rCDI, FMT remains in the experimental phase and should not be considered as a treatment choice outside of research settings. Further controlled trials are required to assess the potential benefits of FMT compared to standard treatment or as an adjunct to standard therapy.

## Conflict of Interest

The authors declare no conflict of interest.

## Author Contributions

S.H., J.Y., and Y.L. contributed equally to this work. S.N., J.C., and D.Y. contributed to the conceptualization of the study. Investigation was carried out by S.N., Y.S., and Z.Y., while methodology was developed by J.C., Y.S., and Z.Y. . Formal analysis was conducted by S.N., D.Y., and J.C. S.N. and Z.Y. were responsible for writing the original draft, and S.N., J.C., and Y.S. participated in reviewing and editing the manuscript.
